# Synthesis, Antifungal Activities and Qualitative Structure Activity Relationship of Carabrone Hydrazone Derivatives as Potential Antifungal Agents

**DOI:** 10.3390/ijms15034257

**Published:** 2014-03-11

**Authors:** Hao Wang, Shuang-Xi Ren, Ze-Yu He, De-Long Wang, Xiao-Nan Yan, Jun-Tao Feng, Xing Zhang

**Affiliations:** Research & Development Center of Biorational Pesticide, Key Laboratory of Plant Protection Resources and Pest Management of Ministry of Education, Northwest A&F University, Xinong Road 22, Yangling 712100, Shaanxi, China; E-Mails: k2wanghao@gmail.com (H.W.); rensx123@gmail.com (S.-X.R.); skyhugh2012@gmail.com (Z.-Y.H.); moraldragonwang@gmail.com (D.-L.W.); orgshylock@gmail.com (X.-N.Y.); zhxing1952@126.com (X.Z.)

**Keywords:** carabrone, hydrazone derivatives, chemical modification, antifungal activity, structure-activity relationship

## Abstract

Aimed at developing novel fungicides for relieving the ever-increasing pressure of agricultural production caused by phytopathogenic fungi, 28 new hydrazone derivatives of carabrone, a natural bioactive sesquisterpene, in three types were designed, synthesized and their antifungal activities against *Botrytis cinerea* and *Colletotrichum lagenarium* were evaluated. The result revealed that all the derivatives synthesized exhibited considerable antifungal activities *in vitro* and *in vivo*, which led to the improved activities for carabrone and its analogues and further confirmed their potential as antifungal agents.

## 1. Introduction

Emerging infectious diseases caused by fungi are increasingly recognized as presenting a worldwide threat to food security, and pathogenic fungi have long been known to constitute a widespread threat to plant species [1,2]. Plant disease epidemics caused by fungi or the fungal-like oomycetes can infect diverse economically important crops and result in severe yield losses and quality reduction of agricultural production [3–5]. Chemical fungicides are widely used to protect crops against these losses at present, but the application of chemical fungicides is challenged by the incidence of resistance and residues [4]. Thus, the discovery of natural antifungal compounds along with further development is important to address these challenges.

**Figure 1.**
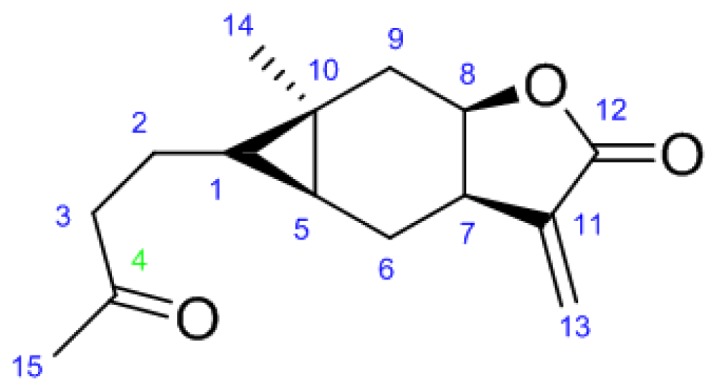
The structure of carabrone.

Carabrone (Figure 1), first isolated from the fruits of *Carpesium abrotanoides* [6], is a well-known sesquiterpene and exhibits significant antibacterial [7,8] and antitumor activities [9,10]. We have demonstrated the antifungal activities of carabrone and its derivatives against *Colletotrichum lagenarium* [11,12] with α-methylene-γ-butyrolactone [13,14] previously, and found the *C*-4 position of carabrone was a key to their activities [11,12]. Besides, as part of an effort to develop environment friendly antifungal agents from natural products, researchers from our laboratory have designed and synthesized a series of carabrone derivatives to explore the qualitative structure–activity relationship (QSAR) hidden in them and the result implied that the derivatives of carabrone with a C=N double bond on the *C*-4 position may display stronger antifungal activity against *C. lagenarium* than others.

**Figure 2.**
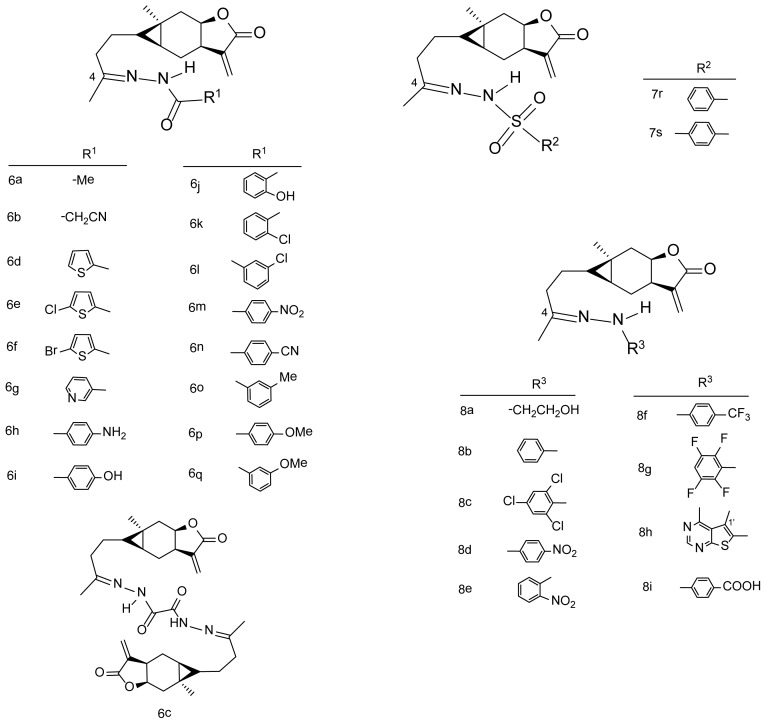
Structures of carabrone hydrazone derivatives.

As a continuation of our project aimed at developing novel fungicides with carabrone as the lead compound, three types of hydrazone derivatives of carabrone were designed in this study by adding a hydrazine or hydrazide group to the *C*-4 site. To our best knowledge, the type of carabrone derivatives formed in sulfonyl hydrazone has not been reported before. Besides, in order to determine whether the carabrone hydrazone derivatives (Figure 2, **6a**–**q**, **7r**–**s**, **8a**–**i**) have the potential for further development, their antifungal activities against *C. lagenarium in vitro* and against *Botrytis cinerea in vitro* and *in vivo* were evaluated [15]. In addition, the QSAR of these compounds were also investigated; it is the first time the QSAR of carabrone derivatives have been obtained based on the experiments both *in vitro* and *in vivo*.

## 2. Results and Discussion

### 2.1. Synthesis

**Scheme 1.**
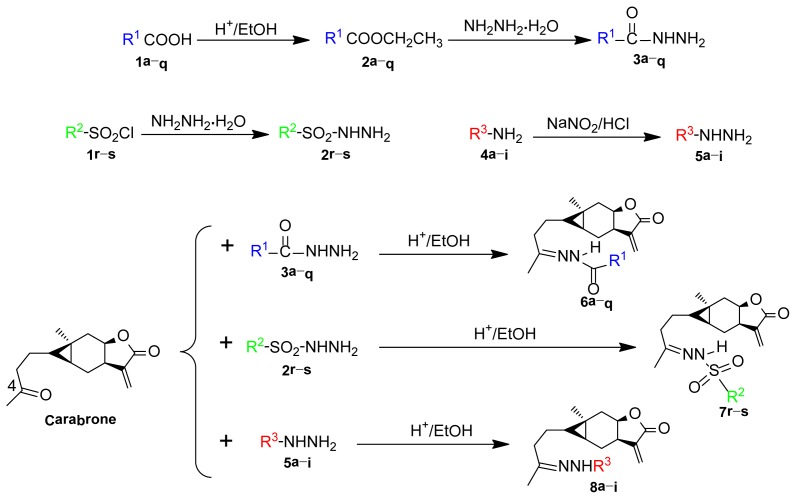
Synthetic route of compounds **6a**–**q**, **7r**–**s** and **8a**–**i**.

Structure modification on active natural compounds is an important way to develop new drugs with improved activities and reduced side effects. Based on our previous finding that antifungal activity can be improved significantly by the introduction of a C=N group to the *C*-4 position of carabrone, catalyzed by glacial acetic acid, 28 new title compounds forming in hydrazone, acyl hydrazone and sulfonyl hydrazone were obtained (Scheme 1) by reacting carabrone with the intermediates prepared prior to use. To our best knowledge, it is the first time to synthesize carabrone derivatives in sulfonyl hydrazone (**7r**–**s**). The structures of all the derivatives were characterized by ^1^H NMR, ^13^C NMR and high-resolution electrospray ionization mass spectrometry (HR-ESI-MS).

### 2.2. Antifungal Activity

All the derivatives exhibited significant antifungal activities in spore germination assay with *IC*_50_ (50% inhibitory concentration, analyzed by SPSS for windows) values ranging from 1.27–27.33 μg/mL against *B. cinerea* and 0.77–15.23 μg/mL against *C. lagenarium* (Table 1). In addition, all derivatives also showed strong antifungal activities against *B. cinerea in vivo* (*IC*_50_ ranged from 2.10–30.80 μg/mL), when testing their inhibition on colonial growth of *B. cinerea* using tomato fruits. Among them, compounds **8g** with the *IC*_50_ value of 1.27 μg/mL against *B. cinerea* and **6q** with 0.77 μg/mL against *C. lagenarium* successively showed the strongest activities to corresponding pathogens *in vitro*, and no significant differences were showed to a commercial fungicide, chlorothalonil. In addition, compound **8c**, with the *IC*_50_ of 2.10 μg/mL against *B. cinerea*, exhibited the strongest activity *in vivo*.

Comparing among carabrone, carabrol and their derivatives [12], it was confirmed that antifungal activity against *C. lagenarium* increased significantly with the introduction of a C=N double bond to the *C*-4 position. Only two compounds as sulfonyl hydrazones of carabrone were designed in our project; they both showed medium antifungal activities. The exact QSAR should be obtained from more derivatives of this type in the future. In addition, it should be especially noted that in our ongoing research, carabrone hydrazone derivatives also showed higher antifungal activities than their oxime ester analogues with the same C=N double bond on the *C*-4 position, both *in vitro* and *in vivo*. This may indicate that the changes in spatial conformation except for the functional group had significant influence on its antifungal activity. In a word, focusing on the lead compound, this study led to an improved activity of carabrone and its analogues and further confirmed their potential as antifungal agents.

### 2.3. Qualitative Structure–Activity Relationship

QSAR analysis based on the activities of hydrazone derivatives of carabrone *in vitro* and *in vivo* would provide some insight into potential rational optimization of active natural compounds to develop new fungicides.

Generally, the introduction of chlorine atom or bromine atom on the substituent group may improve the antifungal activity of the hydrazone derivatives of carabrone, both *in vitro* and *in vivo*. For example, when a chlorine atom was introduced into compound **6d**, affording **6e**, the antifungal activity increased, with the *in vitro* and *in vivo IC*_50_ against *B. cinerea* decreasing from 7.81 and 12.84 μg/mL to 5.57 and 9.57 μg/mL, and with the *in vitro IC*_50_ against *C. lagenarium* decreasing from 10.83–9.98 μg/mL. When a bromine atom was introduced into **6d**, generating **6f**, the *in vitro* antifungal activities against *B. cinerea* and *C. lagenarium* were enhanced, with an *IC*_50_ of 4.83 and 10.02 μg/mL, respectively, along with an improved antifungal activity against *B. cinerea in vivo* at 7.03 μg/mL.

The hydrazone derivatives of carabrone containing meta-substituted R groups showed higher antifungal activities than those derivatives with para-substituted R groups. On the other hand, the antifungal activities of the derivatives with ortho-substituted R groups were lower than those of derivatives with para-substituted R groups. For example, compound **6q** (*in vitro IC*_50_: 6.37 μg/mL against *B. cinerea* and 0.77 μg/mL against *C. lagenarium; in vivo IC*_50_: 12.52 μg/mL against *B. cinerea*) showed stronger antifungal activities than that of **6p** (*in vitro IC*_50_: 10.30 μg/mL against *B. cinerea* and 2.56 μg/mL against *C. lagenarium; in vivo IC*_50_: 16.42 μg/mL against *B. cinerea*). Compound **8e** (*in vitro IC*_50_: 10.31 μg/mL against *B. cinerea* and 4.27 μg/mL against *C. lagenarium; in vivo IC*_50_: 8.93 μg/mL against *B. cinerea*) exhibited a weaker activity than that of compound **8d** (*in vitro IC*_50_: 3.79 μg/mL against *B. cinerea* and 3.95 μg/mL against *C. lagenarium; in vivo IC*_50_: 4.62 μg/mL against *B. cinerea*). In addition, compound **6j** exhibited a weaker activity than that of compound **6i**, following the same rules.

In addition, the hydrazone derivatives of carabrone with a N-containing heterocyclic (**6g**,**8h**) or S-containing heterocyclic (**6d**–**f**) all showed stronger antifungal activities against *B. cinerea in vitro* and *in vivo* than that of the lead compound.

## 3. Experimental Section

### 3.1. General

The melting points of the compounds were determined using an X-4 apparatus (uncorrected, Beijing Tech. Instrument Co., Beijing, China). ^1^H NMR, ^13^C NMR and 2D-NMR spectra were obtained using a Bruker Avance 500 MHz spectrometer (Bruker, Bremerhaven, Germany) in CDCl_3_ or CD_3_COCD_3_ solution with TMS as the internal standard. HR-ESI-MS spectra were carried out using Bruker/Germany apex-ultra 7.0 T spectrometer (Bruker, Bremerhaven, Germany). Carabrone was isolated from *Carpesium macrocephalu* collected in Gansu province, China. Carboxylic acid reagents were purchased from J&K China Chemical Ltd. (Beijing, China). All organic solvents were commercial products and were purified by standard techniques prior to use. Silica gel for TLC and CC was obtained from Qingdao Haiyang Chemical Co. Ltd. (Qingdao, China).

### 3.2. Synthesis of Title Compounds

As shown in Scheme 1, 10 mmol carboxylic acid (**1a**–**q**) was first reacted with excess ethanol in a three-necked, round-bottomed flask containing concentrated sulfuric acid as catalyst, reflux for 5 h, to get the corresponding esters (**2a**–**q**) [16]. The esters were then reacted with excess hydrazine hydrate directly without further purification. The second reaction was performed under reflux for 5–8 h until the reaction was complete as determined by TLC. The intermediates (**3a**–**q**) were isolated by re-crystallization and then reacted with carabrone (100 mg, 0.4 mmol) to obtain the target compounds (**6a**–**q**, Figure 2), catalyzed by glacial acetic acid [17] (See Supplementary for more information).

#### 3.2.1. *N*′-(4-((4a*S*,5*S*,5a*R*)-5a-Methyl-3-methylene-2-oxooctahydro-2*H*-cyclopropa[f]benzofuran- 5-yl)butan-2-ylidene)acetohydrazide (**6a**)

^1^H NMR (500 MHz, CDCl_3_) δ: 9.16 (1H, s), 6.22 (1H, d, *J* = 2.6 Hz), 5.56 (1H, d, *J* = 2.6 Hz), 4.77–4.82 (1H, m), 3.17–3.18 (1H, m), 2.37 (2H, t, *J* = 7.6 Hz), 2.30–2.34 (2H, m), 2.23 (3H, s), 1.88 (3H, s), 1.49–1.64 (2H, m), 1.09 (3H, s), 0.89–1.00 (2H, m), 0.47–0.49 (1H, m), 0.36–0.40 (m, 1H, *H*-5); ^13^C NMR (125 MHz, CDCl_3_) *δ*: 173.7, 170.5, 152.0, 139.0, 122.5, 75.7, 38.8, 37.7, 37.3, 34.4, 30.8, 25.7, 22.9, 20.5, 18.3, 17.1, 15.5; HR-MS (ESI): *m*/*z* calcd for C_17_H_25_N_2_O_3_ ([M + H]^+^), 305.1860; found, 305.1860.

#### 3.2.2. 2-Cyano-*N*′-(4-((4a*S*,5*S*,5a*R*)-5a-methyl-3-methylene-2-oxooctahydro-2*H*-cyclopropa[f]benzofuran- 5-yl)butan-2-ylidene)acetohydrazide (**6b**)

^1^H NMR (500 MHz, CDCl_3_) δ: 10.02 (1H, s), 6.24 (1H, d, *J* = 2.6 Hz), 5.56 (1H, d, *J* = 2.6 Hz), 4.75–4.81 (1H, m), 3.81 (2H, s), 3.12–3.23 (1H, m), 2.38 (2H, t, *J* = 7.6 Hz), 2.25–2.31 (2H, m), 1.95 (3H, s), 1.48–1.63 (2H, m), 1.09 (3H, s), 0.90–1.01 (2H, m), 0.40–0.46 (1H, m), 0.37–0.41 (1H, m); ^13^C NMR (125 MHz, CDCl_3_) *δ*: 170.6, 165.1, 156.0, 139.1, 129.0, 122.5, 75.7, 38.8, 37.5, 37.1, 33.9, 30.5, 25.6, 24.4, 22.8, 18.1, 17.0, 16.2; HR-MS (ESI): *m*/*z* calcd for C_18_H_24_N_3_O_3_ ([M + H]^+^), 330.1812; found, 330.1812.

#### 3.2.3. *N*′1, *N*′2-Bis(4-((4a*S*,5*S*,5a*R*)-5a-methyl-3-methylene-2-oxooctahydro-2*H*-cyclo-propa[f]benzofuran- 5-yl)butan-2-ylidene)oxalohydrazide (**6c**)

^1^H NMR (500 MHz, CDCl_3_) δ: 9.93 (2H, s), 6.23 (2H, d, *J* = 2.6 Hz), 5.56 (2H, d, *J* = 2.6 Hz), 4.85–4.98 (2H, m), 3.11–3.19 (2H, m), 2.49 (4H, t, *J* = 7.6 Hz), 2.30–2.37 (4H, m), 2.01 (6H, s), 1.48–1.75 (4H, m), 1.09 (6H, s), 0.89–1.01 (4H, m), 0.45–0.51 (2H, m), 0.36–0.42 (2H, m); ^13^C NMR (125 MHz, CDCl_3_) *δ*: 170.5, 162.4, 155.2, 139.0, 122.6, 75.6, 39.1, 37.7, 37.2, 34.3, 30.7, 26.1, 23.0, 18.4, 17.3, 15.9; HR-MS (ESI): *m*/*z* calcd for C_32_H_43_N_4_O_6_ ([M + H]^+^), 579.3177; found, 579.3183.

#### 3.2.4. *N*′-(4-((4a*S*,5*S*,5a*R*)-5a-Methyl-3-methylene-2-oxooctahydro-2*H*-cyclopropa[f]-benzofuran- 5-yl)butan-2-ylidene)thiophene-2-carbohydrazide (**6d**)

^1^H NMR (500 MHz, CDCl_3_) δ: 10.39 (1H, s), 8.14 (1H, d, *J* = 3.7 Hz), 7.62 (1H, d, *J* = 4.9 Hz), 7.10 (1H, dd, *J* = 4.9, 3.7 Hz), 6.16 (1H, d, *J* = 2.6 Hz), 5.48 (1H, d, *J* = 2.6 Hz), 4.72–4.77 (1H, m), 3.05–3.14 (1H, m), 2.45–2.48 (2H, t, *J* = 7.1 Hz, *H*-3), 2.25–2.32 (2H, m), 2.05 (3H, s), 1.62–1.74 (2H, m), 1.09 (3H, s), 0.82–0.96 (2H, m), 0.48–0.54 (1H, m), 0.35–0.39 (1H, m); ^13^C NMR (125 MHz, CDCl_3_) *δ*: 170.5, 163.1, 154.0, 139.1, 135.0, 134.5, 133.3, 126.2, 122.4, 75.7, 38.8, 37.6, 37.3, 34.4, 30.7, 26.2, 22.9, 18.3, 17.1, 16.0; HR-MS (ESI): *m*/*z* calcd for C_20_H_25_N_2_O_3_S ([M + H]^+^), 373.1580; found, 373.1581.

#### 3.2.5. 5-Chloro-*N*′-(4-((4a*S*,5*S*,5a*R*)-5a-methyl-3-methylene-2-oxooctahydro-2*H*-cyclopropa[f]benzofuran- 5-yl)butan-2-ylidene)thiophene-2-carbohydrazide (**6e**)

^1^H NMR (500 MHz, CDCl_3_) δ: 9.78 (1H, s), 7.90 (1H, d, *J* = 4.2 Hz), 6.96 (1H, d, *J* = 4.2 Hz), 6.23 (1H, d, *J* = 2.6 Hz), 5.53 (1H, d, *J* = 2.6 Hz), 4.76–4.81 (1H, m), 3.12–3.17 (1H, m), 2.50 (2H, t, *J* = 7.6 Hz), 2.28–2.39 (2H, m), 2.00 (3H, s), 1.49–1.81 (2H, m), 1.14 (3H, s), 0.89–1.00 (2H, m), 0.51–0.56 (1H, m), 0.38–0.43 (1H, m); ^13^C NMR (125 MHz, CDCl_3_) *δ*: 170.5, 162.0, 153.6, 139.9, 139.0, 134.5, 130.2, 125.5, 122.6, 75.7, 38.7, 37.7, 37.3, 34.3, 30.8, 26.3, 23.1, 18.4, 17.2, 15.8; HR-MS (ESI): *m*/*z* calcd for C_20_H_24_ClN_2_O_3_S ([M + H]^+^), 407.1191; found, 407.1191.

#### 3.2.6. 5-Bromo-*N*′-(4-((4a*S*,5*S*,5a*R*)-5a-methyl-3-methylene-2-oxooctahydro-2*H*-cyclo-propa[f]benzofuran- 5-yl)butan-2-ylidene)thiophene-2-carbohydrazide (**6f**)

^1^H NMR (500 MHz, CDCl_3_) δ: 9.86 (1H, s), 7.86 (1H, d, *J* = 4.2 Hz), 7.09 (1H, d, *J* = 4.2 Hz), 6.22 (1H, d, *J* = 2.5 Hz), 5.53 (1H, d, *J* = 2.5 Hz), 4.76–4.81 (1H, m), 3.12–3.18 (1H, m), 2.49 (2H, t, *J* = 7.1 Hz, *H*-3), 2.28–2.39 (2H, m), 2.01 (3H, s), 1.57–1.80 (2H, m), 1.15 (3H, s), 0.89–1.00 (2H, m), 0.51–0.56 (1H, m), 0.38–0.44 (1H, m); ^13^C NMR (125 MHz, CDCl_3_) *δ*: 170.5, 162.0, 153.8, 139.0, 135.1, 133.2, 129.1, 123.6, 122.6, 75.7, 38.7, 37.8, 37.3, 34.3, 30.8, 26.3, 23.1, 18.4, 17.2, 15.9; HR-MS (ESI): *m*/*z* calcd for C_20_H_24_BrN_2_O_3_S ([M + H]^+^), 451.0686; found, 451.0684.

#### 3.2.7. *N*′-(4-((4a*S*,5*S*,5a*R*)-5a-Methyl-3-methylene-2-oxooctahydro-2*H*-cyclopropa[f]-benzofuran- 5-yl)butan-2-ylidene)nicotinohydrazide (**6g**)

^1^H NMR (500 MHz, CDCl_3_) δ: 9.79 (1H, s), 9.02 (1H, s), 8.65 (1H, d, *J* = 6.7 Hz), 8.16 (1H, d, *J* = 7.5 Hz), 7.34 (1H, dd, *J* = 7.5, 6.7 Hz), 6.16 (1H, d, *J* = 2.6 Hz), 5.54 (1H, d, *J* = 2.6 Hz), 4.74–4.79 (1H, m), 3.10–3.17 (1H, m), 2.42 (2H, t, *J* = 7.6 Hz), 2.24–2.29 (2H, m), 2.04 (3H, s), 1.41–1.68 (2H, m), 1.08 (3H, s), 0.85–0.98 (2H, m), 0.31–0.49 (2H, m); ^13^C NMR (125 MHz, CDCl_3_) *δ*: 170.6, 161.6, 152.0, 148.4, 148.3, 139.0, 135.5, 129.7, 123.3, 122.5, 75.7, 39.0, 37.4, 37.0, 34.1, 30.5, 26.2, 22.8, 18.1, 17.0, 14.1; HR-MS (ESI): *m*/*z* calcd for C_21_H_26_N_3_O_3_ ([M + H]^+^), 368.1969; found, 368.1969.

#### 3.2.8. 4-Amino-*N*′-(4-((4a*S*,5*S*,5a*R*)-5a-methyl-3-methylene-2-oxooctahydro-2*H*-cyclo-propa[f]benzofuran- 5-yl)butan-2-ylidene)benzohydrazide (**6h**)

^1^H NMR (500 MHz, CDCl_3_) δ: 9.04 (1H, s), 7.62 (2H, d, *J* = 7.9 Hz), 6.60 (2H, d, *J* = 7.9 Hz), 6.14 (1H, d, *J* = 2.6 Hz), 5.54 (1H, d, *J* = 2.6 Hz), 4.66–4.77 (1H, m), 4.29 (2H, s), 3.07–3.11 (1H, m), 2.42 (2H, t, *J* = 7.6 Hz), 2.25–2.29 (2H, m), 1.95 (3H, s), 1.41–1.66 (2H, m), 1.03 (3H, s), 0.79–0.92 (2H, m), 0.40–0.45 (1H, m), 0.29–0.35 (1H, m); ^13^C NMR (125 MHz, CDCl_3_) *δ*: 170.7, 164.2, 158.0, 150.8, 139.0, 129.2, 122.6, 121.7, 113.8, 75.8, 38.9, 37.4, 37.0, 34.1, 30.5, 26.2, 22.8, 18.2, 17.0, 15.5; HR-MS (ESI): *m*/*z* calcd for C_22_H_28_N_3_O_3_ ([M + H]^+^), 382.2125; found, 382.2124.

#### 3.2.9. 4-Hydroxy-*N*′-(4-((4a*S*,5*S*,5a*R*)-5a-methyl-3-methylene-2-oxooctahydro-2*H*-cyclopropa[f]benzofuran- 5-yl)butan-2-ylidene)benzohydrazide (**6i**)

^1^H NMR (500 MHz, CDCl_3_) δ: 9.24 (1H, s), 7.60 (2H, d, *J* = 7.3 Hz), 6.88 (2H, d, *J* = 7.3 Hz), 6.23 (1H, d, *J* = 2.6 Hz), 5.57 (1H, d, *J* = 2.6 Hz), 4.66–4.74 (1H, m), 3.03–3.11 (1H, m), 2.46 (2H, t, *J* = 7.6 Hz), 2.25–2.30 (2H, m), 2.16 (3H, s), 1.59–1.62 (2H, m), 0.99 (3H, s), 0.79–0.94 (2H, m), 0.29–0.45 (2H, m); ^13^C NMR (125 MHz, CDCl_3_) δ: 171.0, 165.0, 161.0, 155.1, 139.0, 129.3, 123.8, 122.7, 115.8, 75.8, 38.8, 37.7, 37.3, 34.2, 30.7, 26.0, 22.9, 18.2, 17.0, 15.9; HR-MS (ESI): *m*/*z* calcd for C_22_H_27_N_2_O_4_ ([M + H]^+^), 383.1965; found, 383.1963.

#### 3.2.10. 2-Hydroxy-*N*′-(4-((4a*S*,5*S*,5a*R*)-5a-methyl-3-methylene-2-oxooctahydro-2*H*-cyclopropa[f]benzo -furan-5-yl)butan-2-ylidene)benzohydrazide (**6j**)

^1^H NMR (500 MHz, CDCl_3_) δ: 6.86–7.62 (4H, m), 6.21 (1H, d, *J* = 2.6 Hz), 5.55 (1H, d, *J* = 2.6 Hz), 4.74–4.78 (1H, m), 3.13–3.15 (1H, m), 2.46 (2H, t, *J* = 7.6 Hz), 2.23–2.38 (2H, m), 2.01 (3H, s), 1.52–1.74 (2H, m), 1.07 (3H, s), 0.84–0.99 (2H, m), 0.35–0.45 (2H, m); ^13^C NMR (125 MHz, CDCl_3_) *δ*: 170.7, 166.1, 160.3, 139.2, 134.4, 126.3, 122.7, 119.0, 118.5, 114.2, 75.8, 38.9, 37.7, 37.3, 34.3, 30.5, 26.1, 22.9, 18.4, 17.1, 16.0; HR-MS (ESI): *m*/*z* calcd for C_22_H_27_N_2_O_4_ ([M+H]^+^), 383.1965; found, 383.1964.

#### 3.2.11. 2-Chloro-*N*′-(4-((4a*S*,5*S*,5a*R*)-5a-methyl-3-methylene-2-oxooctahydro-2*H*-cyclopropa[f]benzofuran- 5-yl)butan-2-ylidene)benzohydrazide (**6k**)

^1^H NMR (500 MHz, CDCl_3_) δ: 9.33 (1H, s), 7.25–7.63 (4H, m), 6.14 (1H, d, *J* = 2.6 Hz), 5.53 (1H, d, *J* = 2.6 Hz), 4.68–4.78 (1H, m), 3.09–3.15 (1H, m), 2.46 (2H, t, *J* = 7.6 Hz), 2.25–2.31 (2H, m), 1.96 (3H, s), 1.21–1.74 (2H, m), 1.09 (3H, s), 0.74–0.98 (2H, m), 0.20–0.48 (2H, m); ^13^C NMR (125 MHz, CDCl_3_) *δ*: 170.4, 162.6, 159.9, 152.4, 139.0, 131.4, 130.8, 130.4, 130.0, 127.0, 122.4, 75.7, 39.0, 37.5, 37.1, 34.2, 30.6, 26.1, 22.9, 18.3, 17.1, 16.1; HR-MS (ESI): *m*/*z* calcd for C_22_H_26_ClN_2_O_3_ ([M + H]^+^), 401.1627; found, 401.1626.

#### 3.2.12. 3-Chloro-*N*′-(4-((4a*S*,5*S*,5a*R*)-5a-methyl-3-methylene-2-oxooctahydro-2*H*-cyclopropa[f]benzofuran- 5-yl)butan-2-ylidene)benzohydrazide (**6l**)

^1^H NMR (500 MHz, CDCl_3_) δ: 9.09 (1H, s), 7.80 (1H, d, *J* = 7.4 Hz), 7.69 (1H, s), 7.46 (1H, d, *J* = 7.7 Hz), 7.34 (1H, dd, *J* = 7.7, 7.4 Hz), 6.18 (1H, d, *J* = 2.5 Hz), 5.54 (1H, d, *J* = 2.5 Hz), 4.73–4.78 (1H, m), 3.12–3.16 (1H, m), 2.47 (2H, t, *J* = 7.6 Hz), 2.27–2.33 (2H, m), 2.11 (3H, s), 1.47–1.68 (2H, m), 1.07 (3H, s), 0.89–0.99 (2H, m), 0.38–0.45 (2H, m); ^13^C NMR (125 MHz, CDCl_3_) *δ*: 170.5, 162.9, 160.5, 139.1, 135.6, 134.7, 131.6, 129.9, 127.6, 125.5, 122.5, 75.7, 39.0, 37.7, 37.1, 34.2, 30.6, 26.2, 22.9, 18.2, 17.1, 16.0; HR-MS (ESI): *m*/*z* calcd for C_22_H_26_ClN_2_O_3_ ([M + H]^+^), 401.1627; found, 401.1626.

#### 3.2.13. *N*′-(4-((4a*S*,5*S*,5a*R*)-5a-Methyl-3-methylene-2-oxooctahydro-2*H*-cyclopropa[f]-benzofuran- 5-yl)butan-2-ylidene)-4-nitrobenzohydrazide (**6m**)

^1^H NMR (500 MHz, CDCl_3_) δ: 9.21 (1H, s), 7.80 (1H, d, *J* = 7.7 Hz), 7.69 (1H, d, *J* = 7.7 Hz), 7.43 (1H, d, *J* = 7.4 Hz), 7.35 (1H, d, *J* = 7.4 Hz), 6.17 (1H, d, *J* = 2.6 Hz), 5.54 (1H, d, *J* = 2.6 Hz), 4.72–4.76 (1H, m), 3.13–3.14 (1H, m), 2.46 (2H, t, *J* = 7.6 Hz), 2.24–2.31 (2H, m), 2.01 (3H, s), 1.48–1.67 (2H, m), 1.07 (3H, s), 0.84–0.99 (2H, m), 0.42–0.45 (1H, m), 0.29–0.40 (1H, m); ^13^C NMR (125 MHz, CDCl_3_) *δ*: 170.5, 163.0, 160.7, 145.6, 139.1, 134.5, 130.5, 129.8, 125.7, 125.5, 122.5, 75.7, 39.0, 37.6, 37.1, 34.2, 30.5, 26.2, 22.9, 18.2, 17.1, 16.1; HR-MS (ESI): *m*/*z* calcd for C_22_H_26_N_3_O_5_ ([M + H]^+^), 412.1867; found, 412.1866.

#### 3.2.14. 4-Cyano-*N*′-(4-((4a*S*,5*S*,5a*R*)-5a-methyl-3-methylene-2-oxooctahydro-2*H*-cyclopropa[f]benzofuran- 5-yl)butan-2-ylidene)benzohydrazide (**6n**)

^1^H NMR (500 MHz, CDCl_3_) δ: 9.38 (1H, s), 7.96 (2H, d, *J* = 7.7 Hz), 7.76 (2H, d, *J* = 7.7 Hz), 6.18 (1H, d, *J* = 2.6 Hz), 5.55 (1H, d, *J* = 2.6 Hz), 4.74–4.79 (1H, m), 3.15–3.16 (1H, m), 2.46 (2H, t, *J* = 7.6 Hz), 2.26–2.33 (2H, m), 1.52–1.55 (2H, m), 1.08 (3H, s), 0.84–0.99 (2H, m), 0.41–0.46 (2H, m); ^13^C NMR (125 MHz, CDCl_3_) *δ*: 170.6, 161.9, 154.4, 139.0, 137.8, 132.3, 128.2, 122.6, 118.0, 115.0, 75.7, 39.0, 37.7, 37.2, 34.2, 30.7, 26.2, 22.9, 18.2, 17.1, 16.2; HR-MS (ESI): *m*/*z* calcd for C_23_H_26_N_3_O_3_ ([M + H]^+^), 392.1969; found, 392.1965.

#### 3.2.15. 3-Methyl-*N*′-(4-((4a*S*,5*S*,5a*R*)-5a-methyl-3-methylene-2-oxooctahydro-2*H*-cyclopropa[f]benzofuran- 5-yl)butan-2-ylidene)benzohydrazide (**6o**)

^1^H NMR (500 MHz, CDCl_3_) δ: 9.16 (1H, s), 7.63 (1H, d, *J* = 7.9 Hz), 7.61 (1H, dd, *J* = 8.1, 7.9 Hz), 7.38 (1H, s), 7.28 (1H, d, *J* = 8.1 Hz), 6.16 (1H, d, *J* = 2.6 Hz), 5.53 (1H, d, *J* = 2.6 Hz), 4.71–4.76 (1H, m), 3.82 (3H, s), 3.10–3.14 (1H, m), 2.44 (2H, t, *J* = 7.6 Hz), 2.37 (3H, s), 2.26–2.31 (2H, m), 2.01 (3H, s), 1.46–1.61 (2H, m), 1.07 (3H, s), 0.89–0.95 (2H, m), 0.37–0.45 (2H, m); ^13^C NMR (125 MHz, CDCl_3_) *δ*: 170.5, 164.2, 159.3, 139.1, 138.3, 133.7, 132.3, 128.3, 128.0, 124.2, 122.4, 75.7, 39.0, 37.5, 37.1, 34.1, 30.5, 26.2, 22.9, 21.2, 18.3, 17.0, 15.8; HR-MS (ESI): *m*/*z* calcd for C_23_H_29_N_2_O_3_ ([M + H]^+^), 381.2173; found, 381.2172.

#### 3.2.16. 4-Methoxy-*N*′-(4-((4a*S*,5*S*,5a*R*)-5a-methyl-3-methylene-2-oxooctahydro-2*H*-cyclopropa[f]benzofuran- 5-yl)butan-2-ylidene)benzohydrazide (**6p**)

^1^H NMR (500 MHz, CDCl_3_) δ: 8.78 (1H, s), 7.81 (2H, d, *J* = 7.7 Hz), 6.93 (2H, d, *J* = 7.7 Hz), 6.22 (1H, d, *J* = 2.6 Hz), 5.54 (1H, d, *J* = 2.6 Hz), 4.74–4.79 (1H, m), 3.85 (3H, s), 3.12–3.18 (1H, m), 2.48 (2H, t, *J* = 7.6 Hz), 2.28–2.35 (2H, m), 2.16 (3H, s), 1.52–1.61 (2H, m), 1.09 (3H, s), 0.92–0.97 (2H, m), 0.37–0.47 (2H, m); ^13^C NMR (125 MHz, CDCl_3_) *δ*: 170.4, 162.4, 160.2, 156.4, 139.0, 129.2, 125.8, 122.5, 113.9, 75.6, 55.4, 39.0, 37.7, 37.3, 34.2, 30.7, 26.2, 22.9, 18.2, 17.2, 15.4; HR-MS (ESI): *m*/*z* calcd for C_23_H_29_N_2_O_4_ ([M + H]^+^), 397.2122; found, 397.2121.

#### 3.2.17. 3-Methoxy-*N*′-(4-((4a*S*,5*S*,5a*R*)-5a-methyl-3-methylene-2-oxooctahydro-2*H*-cyclopropa[f]benzofuran- 5-yl)butan-2-ylidene)benzohydrazide (**6q**)

^1^H NMR (500 MHz, CDCl_3_) δ: 9.03 (1H, s), 7.29–7.37 (3H, m), 7.03 (1H, s), 6.18 (1H, d, *J* = 2.6 Hz), 5.54 (1H, d, *J* = 2.6 Hz), 4.73–4.78 (1H, m), 3.82 (3H, s), 3.13–3.15 (1H, m), 2.48 (2H, t, *J* = 7.6 Hz), 2.26–2.31 (2H, m), 2.14 (3H, s), 1.51–1.61 (2H, m), 1.07 (3H, s), 0.89–0.96 (2H, m), 0.38–0.46 (2H, m); ^13^C NMR (125 MHz, CDCl_3_) *δ*: 170.5, 163.8, 159.7, 153.6, 139.0, 135.1, 129.6, 122.5, 119.0, 117.7, 112.8, 75.7, 55.4, 39.0, 37.6, 37.1, 34.2, 30.6, 26.2, 22.9, 18.2, 17.1, 15.8; HR-MS (ESI): *m*/*z* calcd for C_23_H_29_N_2_O_4_ ([M + H]^+^), 397.2122; found, 397.2121.

For the preparation of carabrone sulfonyl hydrazone (**7r**–**s**), a solution (20 mL) of benzenesulfonyl chloride (**1r**, 1 mmol) or *p*-toluene sulfonyl chloride (**1s**, 1 mmol) in acetone and an appropriate amount of hydrazine hydrate were treated with 5% NaOH solution (0.5 mL) firstly. The mixture was then shaken vigorously for 10 min, cooled and poured into 1:1 (*v*/*v*) HCl. The precipitate formed was filtered, washed with water and recrystallized from alcohol to obtain the intermediates (**2r**–**s**) [18]. Finally, the intermediates (0.5 mmol) was added to a three-necked, round-bottomed flask containing carabrone (100 mg, 0.4 mmol) in absolute ethyl alcohol, reflux until the reaction was complete, catalyzed by glacial acetic acid [17] as shown in Scheme 1.

#### 3.2.18. *N*′-((*E*)-4-((4a*S*,5*S*,5a*R*)-5a-Methyl-3-methylene-2-oxooctahydro-2*H*-cyclopropa[f]benzofuran- 5-yl)butan-2-ylidene)benzenesulfonohydrazide (**7r**)

^1^H NMR (500 MHz, CDCl_3_) δ: 7.96 (2H, d, *J* = 8.7 Hz), 7.45–7.58 (3H, m), 6.22 (1H, d, *J* = 2.5 Hz), 5.56 (1H, d, *J* = 2.5 Hz), 4.70–4.75 (1H, m), 3.07–3.13 (1H, m), 2.48 (2H, t, *J* = 7.6 Hz), 2.26–2.31 (2H, m), 1.22–1.37 (2H, m), 0.99 (3H, s), 0.78–0.91 (2H, m), 0.21–0.39 (*H*-1, m); ^13^C NMR (125 MHz, CDCl_3_) *δ*: 170.8, 158.8, 139.0, 138.4, 133.1, 128.8, 128.0, 122.7, 75.7, 38.5, 37.6, 37.1, 33.8, 30.5, 25.4, 22.9, 18.2, 16.8, 16.1; HR-MS (ESI): *m*/*z* calcd for C_21_H_27_N_2_O_4_S ([M + H]^+^), 403.1686; found, 403.1689.

#### 3.2.19. 4-Methyl-*N*′-((*E*)-4-((4a*S*,5*S*,5a*R*)-5a-methyl-3-methylene-2-oxooctahydro-2*H*-cyclopropa[f]benzofuran-5-yl)butan-2-ylidene)benzenesulfonohydrazide (**7s**)

^1^H NMR (500 MHz, CDCl_3_) δ: 7.74 (2H, d, *J* = 7.9 Hz), 7.29 (2H, d, *J* = 7.9 Hz), 6.24 (1H, d, *J* = 2.6 Hz), 5.56 (1H, d, *J* = 2.6 Hz), 4.71–4.75 (1H, m), 3.09–3.18 (1H, m), 2.40 (2H, t, *J* = 7.6 Hz), 2.24–2.30 (2H, m), 1.79 (3H, s), 1.51–1.65 (2H, m), 1.26 (3H, s), 1.02 (3H, s), 0.81–0.97 (2H, m), 0.28–0.47 (2H, m); ^13^C NMR (125 MHz, CDCl_3_) *δ*: 170.5, 158.1, 144.0, 139.1, 135.5, 129.4, 128.1, 122.5, 75.6, 38.5, 37.7, 37.2, 34.0, 30.6, 25.5, 23.0, 21.5, 18.2, 17.0, 16.0; HR-MS (ESI): *m*/*z* calcd for C_22_H_29_N_2_O_4_S ([M + H]^+^), 417.1843; found, 417.1845.

As shown in Scheme 1, for the preparation of the intermediates (**5a**–**i**), 2-hydroxyl ethanamine (**4a**, 1 mmol) or phenylamine derivatives (**4b**–**i**, 1 mmol) was first reacted with sodium nitrite (2 mmol) and hydrochloric acid (5 mL) at 0 °C, reduced by sodium pyrosulfite, washed with hydrochloric acid (5 mL) and dissolved in water (10 mL). Concentrated hydrochloric acid was then added to this solution after decolorization with activated carbon to produce intermediates (**5a**–**i**) [19]. Finally, the target compounds (**8a**–**i**, Figure 2) were successively obtained with the yields of 45%–93% after carabrone (100 mg, 0.4 mmol) reacting with the intermediates (0.5 mmol) and catalyzed by glacial acetic acid [17].

#### 3.2.20. (4a*S*,5*S*,5a*R*)-5-(3-(2-(2-Hydroxyethyl)hydrazono)butyl)-5a-methyl-3-methyl-eneoctahydro- 2*H*-cyclopropa[f]benzofuran-2-one (**8a**)

^1^H NMR (500 MHz, CDCl_3_) δ: 6.24 (1H, d, *J* = 2.6 Hz), 5.5(1H, d, *J* = 2.6 Hz), 4.74–4.81 (1H, m), 3.82 (2H, t, *J* = 4.6 Hz), 3.72 (1H, s), 3.27 (2H, t, *J* = 4.6 Hz), 3.13–3.19 (1H, m), 2.40 (2H, t, *J* = 7.6 Hz), 2.24–2.30 (2H, m), 2.17 (3H, s), 1.48–1.63 (2H, m), 1.09 (3H, s), 0.86–0.98 (2H, m), 0.28–0.46 (2H, m); ^13^C NMR (125 MHz, CDCl_3_) *δ*: 170.4, 150.0, 139.1, 122.5, 75.6, 63.7, 51.9, 38.8, 37.7, 37.3, 34.3, 30.7, 23.4, 23.0, 18.2, 17.2, 14.5; HR-MS (ESI): *m*/*z* calcd for C_17_H_27_N_2_O_3_ ([M + H]^+^), 307.1940; found, 307.1938.

#### 3.2.21. (4a*S*,5*S*,5a*R*)-5a-Methyl-3-methylene-5-(3-(2-phenylhydrazono)butyl)-octa-hydro-2*H*-cyclopropa[ f]benzofuran-2-one (**8b**)

^1^H NMR (500 MHz, CDCl_3_) δ: 7.35–7.47 (6H, m), 6.22 (1H, d, *J* = 2.5 Hz), 5.54 (1H, d, *J* = 2.5 Hz), 4.75–4.77 (1H, m), 3.09–3.17 (1H, m), 2.44 (2H, t, *J* = 7.6 Hz), 2.25–2.31 (2H, m), 1.55 (s, 3H, *H*-15), 1.22–1.45 (2H, m), 1.06 (3H, s), 0.88–0.96 (2H, m), 0.33–0.47 (2H, m); ^13^C NMR (125 MHz, CDCl_3_) *δ*: 170.7, 151.0, 139.1, 131.4, 129.2, 122.6, 122.5, 104.9, 75.8, 38.5, 37.7, 37.3, 34.2, 30.8, 26.3, 22.9, 18.2, 17.2, 15.7; HR-MS (ESI): *m*/*z* calcd for C_21_H_27_N_2_O_2_([M+H]^+^), 339.2067; found, 339.2068.

#### 3.2.22. (4a*S*,5*S*,5a*R*)-5a-Methyl-3-methylene-5-(3-(2-(2,4,6-trichlorophenyl)-hydrazono)butyl)- octahydro-2*H*-cyclopropa[f]benzofuran-2-one (**8c**)

^1^H NMR (500 MHz; CDCl_3_) δ: 9.21 (1H; s); 7.42 (2H; s); 6.23 (1H,d; *J* = 2.6 Hz); 5.55 (1H; d; *J* = 2.6 Hz); 4.76–4.81 (1H; m); 3.13–3.19 (1H; m); 2.46 (2H; t; *J* = 7.6 Hz); 2.25–2.31 (2H; m); 1.62 (3H; s); 1.41–1.68 (2H; m); 1.10 (3H; s); 0.87–1.00 (2H; m); 0.36–0.52 (2H; m); ^13^C NMR (125 MHz; CDCl_3_) *δ*: 170.6; 153.3; 145.5; 139.1; 128.7; 127.0; 122.5; 75.7; 38.6; 37.8; 37.4; 34.5; 30.8; 26.1; 23.0; 18.3; 17.2; 15.1; HR-MS (ESI): *m/z* calcd for C_21_H_24_Cl_3_N_2_O_2_ ([M + H]^+^); 441.0898; found; 441.0896.

#### 3.2.23. (4a*S*,5*S*,5a*R*)-5a-Methyl-3-methylene-5-(3-(2-(4-nitrophenyl)hydrazono)-butyl)-octahydro-2*H-*cyclopropa[ f]benzofuran-2-one (**8d**)

^1^H NMR (500 MHz, CDCl_3_) δ: 8.12 (2H, d, *J* = 8.4 Hz), 7.74 (1H, s), 7.06 (2H, d, *J* = 8.4 Hz), 6.21 (1H, d, *J* = 2.6 Hz), 5.54 (1H, d, *J* = 2.6 Hz), 4.78–4.80 (1H, m), 3.16–3.18 (1H, m), 2.42 (2H, t, *J* = 7.6 Hz), 2.26–2.35 (2H, m), 1.93 (3H, s), 1.56–1.69 (2H, m), 1.11 (3H, s), 0.92–1.03 (2H, m), 0.48–0.52 (1H, m), 0.39–0.43 (1H, m); ^13^C NMR (125 MHz, CDCl_3_) *δ*: 170.7, 150.7, 150.5, 139.6, 139.1, 126.1, 122.7, 111.6, 75.8, 38.9, 37.6, 37.3, 34.3, 30.7, 26.0, 23.0, 18.4, 17.2, 15.0; HR-MS (ESI): *m*/*z* calcd for C_21_H_26_N_3_O_4_ ([M + H]^+^), 384.1918; found, 384.1917.

#### 3.2.24. (4a*S*,5*S*,5a*R*)-5a-Methyl-3-methylene-5-(3-(2-(2-nitrophenyl)hydrazono)butyl)-octahydro-2*H-*cyclopropa[ f]benzofuran-2-one (**8e**)

^1^H NMR (500 MHz, CDCl_3_) δ: 10.67 (1H, s), 8.15 (1H, d, *J* = 8.7 Hz), 7.51–7.57 (2H, m), 6.77 (1H, d, *J* = 8.0 Hz), 6.22 (1H, d, *J* = 2.6 Hz), 5.52 (1H, d, *J* = 2.6 Hz), 4.76–4.79 (1H, m), 3.15–3.17 (1H, m), 2.48 (2H, t, *J* = 7.7 Hz), 2.28–2.38 (2H, m), 2.01 (3H, s), 1.52–1.78 (2H, m), 1.12 (3H, s), 0.92–1.01 (2H, m), 0.50–0.55 (1H, m), 0.37–0.42 (1H, m); ^13^C NMR (125 MHz, CDCl_3_) *δ*: 170.5, 152.8, 142.6, 139.1, 136.2, 130.7, 125.9, 122.5, 117.5, 115.8, 75.6, 39.0, 37.7, 37.4, 34.6, 30.8, 26.1, 23.0, 18.4, 17.2, 15.8; HR-MS (ESI): *m*/*z* calcd for C_21_H_26_N_3_O_4_ ([M + H]^+^), 384.1918; found, 384.1917.

#### 3.2.25. (4a*S*,5*S*,5a*R*)-5a-Methyl-3-methylene-5-(3-(2-(4-(trifluoromethyl)phenyl)-hydrazono)butyl)- octahydro-2*H*-cyclopropa[f]benzofuran-2-one (**8f**)

^1^H NMR (500 MHz, CDCl_3_) δ: 7.42 (2H, d, *J* = 8.3 Hz), 7.09 (2H, d, *J* = 8.3 Hz), 6.18 (1H, d, *J* = 2.6 Hz), 5.49 (1H, d, *J* = 2.6 Hz), 4.74–4.76 (1H, m), 3.11–3.13 (1H, m), 2.39 (2H, t, *J* = 7.6 Hz), 2.23–2.31 (2H, m), 1.86 (3H, s), 1.51–1.67 (2H, m), 1.08 (3H, s), 0.86–0.98 (2H, m), 0.45–0.50 (1H, m), 0.34–0.39 (1H, m); ^13^C NMR (125 MHz, CDCl_3_) *δ*: 170.7, 153.0, 148.6, 148.1, 139.2, 126.4, 122.8, 122.5, 112.2, 75.9, 38.8, 37.6, 37.3, 34.5, 30.8, 26.1, 22.9, 18.2, 17.1, 14.8; HR-MS (ESI): *m*/*z* calcd for C_22_H_26_F_3_N_2_O_2_ ([M + H]^+^), 407.1941; found, 407.1932.

#### 3.2.26. (4a*S*,5*S*,5a*R*)-5a-Methyl-3-methylene-5-(3-(2-(2,3,5,6-tetrafluorophenyl)-hydrazono)butyl)- octahydro-2*H*-cyclopropa[f]benzofuran-2-one (**8g**)

^1^H NMR (500 MHz, CDCl_3_) δ: 6.78 (1H, s), 6.20 (1H, d, *J* = 2.5 Hz), 5.55 (1H, d, *J* = 2.5 Hz), 4.76–4.81 (1H, m), 3.16–3.20 (1H, m), 2.46 (2H, t, *J* = 7.6 Hz), 2.26–2.33 (2H, m), 1.94 (s, 3H, *H*-15), 1.49–1.69 (2H, m), 1.10 (3H, s), 0.88–1.00 (2H, m), 0.45–0.54 (1H, m), 0.36–0.42 (1H, m); ^13^C NMR (125 MHz, CDCl_3_) *δ*: 170.6, 153.9, 147.4, 145.4, 139.2, 126.7, 122.3, 96.3, 75.7, 38.5, 37.6, 37.2, 34.4, 30.7, 25.9, 22.9, 18.0, 17.1, 14.7; HR-MS (ESI): *m*/*z* calcd for C_21_H_23_F_4_N_2_O_2_ ([M + H]^+^), 411.1690; found, 411.1689.

#### 3.2.27. (4a*S*,5*S*,5a*R*)-5-(3-(2-(5,6-Dimethylthieno[2,3-d]pyrimidin-4-yl)hydrazono)-butyl)-5a-methyl- 3-methyleneoctahydro-2*H*-cyclopropa[f]benzofuran-2-one (**8h**)

^1^H NMR (500 MHz, CDCl_3_) δ: 8.42 (1H, s), 6.21 (1H, d, *J* = 2.5 Hz), 5.53 (1H, d, *J* = 2.5 Hz), 4.76–4.81 (1H, m), 3.12–3.18 (1H, m), 2.51 (3H, s), 2.44 (2H, t, *J* = 7.6 Hz), 2.40 (3H, s), 2.28–2.32 (2H, m), 2.07 (3H, s), 1.52–1.72 (2H, m), 1.09 (3H, s), 0.93–1.03 (2H, m), 0.46–0.51 (1H, m), 0.36–0.41 (1H, m); ^13^C NMR (125 MHz, CDCl_3_) *δ*: 170.4, 165.0, 157.4, 152.3, 139.1, 130.9, 130.8, 123.4, 122.5, 116.8, 75.6, 39.0, 37.6, 37.2, 34.4, 30.7, 26.6, 23.0, 18.4, 17.2, 15.5, 14.2, 13.2; HR-MS (ESI): *m*/*z* calcd for C_23_H_29_N_4_O_2_S ([M + H]^+^), 425.2006; found, 425.2004.

#### 3.2.28. 4-(2-(4-((4a*S*,5*S*,5a*R*)-5a-Methyl-3-methylene-2-oxooctahydro-2*H*-cyclopropa[f]benzofuran- 5-yl)butan-2-ylidene)hydrazinyl)benzoic acid (**8i**)

^1^H NMR (500 MHz, (CD_3_)_2_CO) δ: 10.86 (1H, s), 8.20 (2H, d, *J* = 8.1 Hz), 7.85 (2H, d, *J* = 8.1 Hz), 6.07 (1H, d, *J* = 2.6 Hz), 5.61 (1H, d, *J* = 2.6 Hz), 4.80–4.86 (1H, m), 3.20–3.27 (1H, m), 2.40 (2H, t, *J* = 7.6 Hz), 2.24–2.33 (2H, m), 1.51–1.59 (2H, m), 1.46 (3H, s), 1.10 (3H, s), 0.89–1.03 (2H, m), 0.52–0.58 (1H, m), 0.40–0.45 (1H, m); ^13^C NMR (125 MHz, (CD_3_)_2_CO) *δ*: 170.7, 150.7, 150.5, 139.6, 139.1, 126.1, 122.7, 111.6, 75.8, 38.9, 37.6, 37.3, 34.3, 30.7, 26.0, 23.0, 18.4, 17.2, 15.0; HR-MS (ESI): *m*/*z* calcd for C_22_H_27_N_2_O_4_ ([M + H]^+^), 383.1965; found, 383.1966.

### 3.3. Microorganism and Preparation of Spore Suspension

The fungal pathogens *C. lagenarium* (Accession No. 36199) and *B. cinerea* were provided by Agricultural Culture Collection of China and the Institute of Plant Disease (Beijing, China), Northwest A&F University (Yangling, China), respectively. *C. lagenarium* was cultured for 2 weeks at 25 °C on potato dextrose agar (PDA, Difco) while *B. cinerea* was cultured at 20 °C on the same medium after being retrieved from the storage tube. Plates were then flooded with sterile distilled water, and the conidia were scraped with a glass rod. Mycelial debris was removed by filtrating through double-layer cheesecloth. The resulting spores were harvested and suspended in sterile distilled water containing 0.1% (*v*/*v*) Tween 20. Concentration of the spore suspension was adjusted to 1.0 × 10^6^ spore/mL by diluting with sterilized distilled water using a SUPERIOR hemocytometer (Marienfeld, Berlin, Germany) [12,20].

### 3.4. Spore Germination Assay

The tested samples (10 mg) dissolved in acetone (0.1 mL) were diluted with sterile distilled water to get the test solutions, with the final concentration of acetone lower than 1% (*v*/*v*) [20]. A series of concentrations of tested samples and negative control (1% acetone with sterile distilled water) were tested on spore germination of *C. lagenarium* or *B. cinerea*. The samples were inoculated with spore suspension of *C. lagenarium* or *B. cinerea* containing 1.0 × 10^6^ spores/mL. Aliquots of 10 μL prepared spore suspension were placed on separate glass slides in triplicate. Slides containing the spores were incubated in a moisture chamber at 25 °C for 6–8 h. Each slide was then checked under the microscope for spore germination. Spores were considered to be germinated when the length of the germ tube reached to at least half of the spore length. The numbers of generated spores were counted, and the percentage of germinated spores was calculated [12]. Chlorothalonil, purchased from Xiangtan Huayuan Fine-Chem Co. Ltd. (Xiangtan, China), was used as the positive control.

### 3.5. Preparation of Tested Tomato Fruits

Tomato fruits, avoiding pesticides for more than one month, were harvested from greenhouse at the mature green stage (mature but fruit surface was green). Harvest fruits were then sorted based on their size and colour. Selected fruits were washed with water, air dried and the surface was then sterilized with sodium hypochlorite solution (1.0 g/L) for 5 min, rinsed twice by sterile distilled water and air dried [21].

### 3.6. Culture of B. cinerea and Inoculation

The *B. cinerea* strain was cultured on PDA at 25 °C for a week. Two symmetrical spots (5 mm in diameter and 3 mm deep) were punctured on the opposite side of tomato fruits with a sterile nail, tested samples were then sprayed on the surface and air dried. Twenty-four hours later, agar discs with mycelium (4 mm in diameter), taking from the edge of 7-day old colonies of *B. cinerea* on PDA, were placed on the punctured site of the tomato fruits with mycelia facing the surface of fruits. Every test was performed in triplicate. Chlorothalonil was used as the positive control. Treated fruits were put in plastic boxes containing sterile water to keep humidity and stored at 25 °C. After 6 days, lesion diameters were measured in two perpendicular directions and the average inhibition rate was calculated [21].

### 3.7. Statistical Analysis

All experimental data were calculated and analyzed using SPSS 16.0 for Windows (SPSS China, Shanghai, China).

## 4. Conclusions

Twenty-eight new hydrazone derivatives of carabrone were synthesized in this work, and most of them exhibited higher antifungal activities against *C. lagenarium* than the lead compound and their ester analogues [12]. The additional experiment carried out against *B. cinerea in vitro* and *in vivo* confirmed their promising potential for development of activities. It is worth noting that **8c** and **8g** exhibited the strongest antifungal activities among these compounds and have been identified as potential candidate compounds for the development of new fungicides for the sustainable agriculture. Given this interesting level of activity, further study on these compounds is necessary.

## Supplementary Information



## Figures and Tables

**Table 1.** Chemical yields and antifungal activities of the 28 hydrazone derivatives of carabrone.

Compound No.	Yield a (%)	*In vitro* (*IC*_50_ b, μg/mL)		*In vivo* (*IC*_50_ b, μg/mL)
	
		*B. cinerea*	*C. lagenarium*	*B. cinerea*
**6a**	78	27.33 ± 1.29	10.18 ± 1.02	29.62 ± 3.12
**6b**	82	9.77 ± 0.68	8.14 ± 0.34	7.55 ± 0.68
**6c**	93	22.07 ± 0.70	10.22 ± 0.62	30.80 ± 1.51
**6d**	78	7.81 ± 0.37	10.83 ± 1.79	12.84 ± 0.31
**6e**	87	5.57 ± 0.89	9.98 ± 0.30	9.57 ± 0.99
**6f**	72	4.83 ± 0.41	10.02 ± 0.72	7.03 ± 0.58
**6g**	70	8.50 ± 0.69	2.06 ± 0.86	4.02 ± 0.26
**6h**	78	2.67 ± 0.10	2.10 ± 0.19	4.85 ± 0.52
**6i**	59	3.46 ± 0.39	1.24 ± 0.47	4.73 ± 1.13
**6j**	48	13.16 ± 0.23	2.01 ± 0.34	13.44 ± 1.02
**6k**	77	3.35 ± 0.65	3.52 ± 0.21	9.67 ± 0.95
**6l**	78	2.57 ± 0.12	1.97 ± 0.78	8.76 ± 1.08
**6m**	61	3.39 ± 0.56	5.49 ± 0.73	6.67 ± 0.49
**6n**	88	2.47 ± 0.72	1.69 ± 0.54	4.29 ± 0.51
**6o**	65	9.00 ± 1.09	0.98 ± 0.19	18.08 ± 0.91
**6p**	53	10.30 ± 0.86	2.56 ± 0.32	16.42 ± 1.24
**6q**	47	6.37 ± 0.71	0.77 ± 0.27	12.52 ± 0.96
**7r**	66	13.32 ± 0.87	6.43 ± 0.95	17.46 ± 0.88
**7s**	73	12.99 ± 0.62	5.33 ± 0.89	17.26 ± 1.05
**8a**	58	17.37 ± 0.91	15.23 ± 1.14	16.69 ± 2.37
**8b**	47	16.32 ± 0.58	7.62 ± 0.19	18.85 ± 1.25
**8c**	75	1.51 ± 0.73	1.53 ± 0.46	2.10 ± 0.47
**8d**	65	3.79 ± 0.82	3.95 ± 0.28	4.62 ± 0.29
**8e**	77	10.31 ± 1.31	4.27 ± 0.35	8.93 ± 0.96
**8f**	72	1.99 ± 0.34	4.05 ± 0.57	5.52 ± 0.37
**8g**	63	1.27 ± 0.16	2.65 ± 0.91	2.59 ± 0.63
**8h**	81	13.32 ± 0.87	1.63 ± 0.45	11.46 ± 1.09
**8i**	45	14.77 ± 0.59	7.06 ± 0.77	14.59 ± 0.95
Carabrone		14.14 ± 0.94	8.29 ± 0.51	16.74 ± 1.32
Chlorothalonil c		0.49 ± 0.28	0.52 ± 0.17	1.29 ± 0.33

a chemical yield at the final step of the synthesis;

b *IC*_50_ represents 50% inhibitory concentration that are presented as the means ± SD (*n* = 3), μg/mL;

c positive control.
