# Supplementary material for: Synthesis, Antifungal Activities and Qualitative Structure Activity Relationship of Carabrone Hydrazone Derivatives as Potential Antifungal Agents
Source: Int J Mol Sci. 2014 Mar 11;15(3):4257–72. doi: 10.3390/ijms15034257 (PMC3975396; doi:10.3390/ijms15034257)
Supplement: Supplementary file 1 [file ijms-15-04257-s001.pdf]

Supplementary Information

Physical Property and Spectroscopic Data of 28 Title Compounds

*1. **6a**: N'-(4-((4aS,5S,5aR)-5a-methyl-3-methylene-2-oxooctahydro-2H-cyclopropa[f]benzofuran-5-yl) butan-2-ylidene)acetohydrazide*

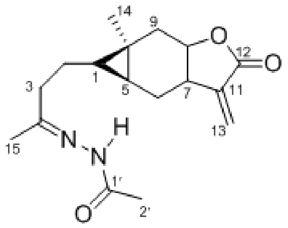

Light yellow solid, mp: 71–72 ℃ ; ^{1}H NMR (500 MHz, $CDCl_3$) δ: 9.16 (s, 1H, NH), 6.22 (d, J = 2.6 Hz, 1H, H-13α), 5.56 (d, J = 2.6 Hz, 1H, H-13β), 4.77–4.82 (m, 1H, H-8), 3.17–3.18 (m, 1H, H-7), 2.37 (t, J = 7.6 Hz, 2H, H-3), 2.30–2.34 (m, 2H, overlapped, H_{eq}-6, H_{eq}-9), 2.23 (s, 3H, H-2'), 1.88 (s, 3H, H-15), 1.49–1.64 (m, 2H, H-2), 1.09 (s, 3H, H-14), 0.89–1.00 (m, 2H, overlapped, H_{eq}-6, H_{eq}-9), 0.47–0.49 (m, 1H, H-1), 0.36–0.40 (m, 1H, H-5); ^{13}C NMR (125 MHz, $CDCl_3$) δ: 173.7 (C-1'), 170.5 (C-12), 152.0 (C-4), 139.0 (C-11), 122.5 (C-13), 75.7 (C-8), 38.8 (C-3), 37.7 (C-7), 37.3 (C-9), 34.4 (C-1), 30.8 (C-6), 25.7 (C-2), 22.9 (C-5), 20.5 (C-2'), 18.3 (C-14), 17.1 (C-10), 15.5 (C-15); HR-MS (ESI): m/z calcd for $C_{17}H_{25}N_2O_3$([M + H]$^+$), 305.1860; found, 305.1860.

*2. **6b**: 2-Cyano-N'-(4-((4aS,5S,5aR)-5a-methyl-3-methylene-2-oxooctahydro-2H-cyclopropa[f] benzofuran-5-yl)butan-2-ylidene)acetohydrazide*

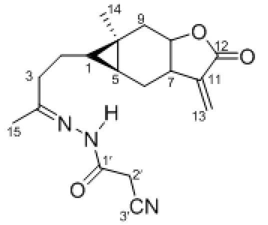

Colourless solid, mp: 79–81 ℃ ; ^{1}H NMR (500 MHz, $CDCl_3$) δ: 10.02 (s, 1H, NH), 6.24 (d, J = 2.6 Hz, 1H, H-13α), 5.56 (d, J = 2.6 Hz, 1H, H-13β), 4.75–4.81 (m, 1H, H-8), 3.81 (s, 2H, H-2'), 3.12–3.23 (m, 1H, H-7), 2.38 (t, J = 7.6 Hz, 2H, H-3), 2.25–2.31 (m, 2H, overlapped, H_{eq}-6, H_{eq}-9),1.95 (s, 3H, H-15), 1.48–1.63 (m, 2H, H-2), 1.09 (s, 3H, H-14), 0.90–1.01 (m, 2H, overlapped, H_{eq}-6, H_{eq}-9), 0.40–0.46 (m, 1H, H-1), 0.37–0.41 (m, 1H, H-5); ^{13}C NMR (125 MHz, $CDCl_3$) δ: 170.6 (C-12), 165.1 (C-1'), 156.0 (C-4), 139.1 (C-11), 129.0 (C-3'), 122.5 (C-13), 75.7 (C-8), 38.8 (C-3), 37.5 (C-7), 37.1 (C-9), 33.9 (C-1), 30.5 (C-6), 25.6 (C-2), 24.4 (C-2'), 22.8 (C-5), 18.1 (C-14), 17.0 (C-10), 16.2 (C-15); HR-MS (ESI): m/z calcd for $C_{18}H_{24}N_3O_3$([M + H]$^+$), 330.1812; found, 330.1812.

3. ***6c****: N'1,N'2-bis(4-((4aS,5S,5aR)-5a-methyl-3-methylene-2-oxooctahydro-2H-cyclopropa[f] benzofuran-5-yl)butan-2-ylidene)oxalohydrazide*

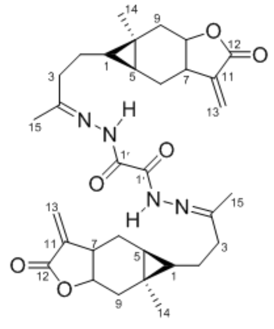

Light yellow solid, mp: 179–180 ℃ ; ^{1}H NMR (500 MHz, $CDCl_3$) δ: 9.93 (s, 2H, NH), 6.23 (d, J = 2.6 Hz, 2H, *H*-13α), 5.56 (d, J = 2.6 Hz, 2H, *H*-13β), 4.85–4.98 (m, 2H, *H*-8), 3.11–3.19 (m, 2H, *H*-7), 2.49 (t, J = 7.6 Hz, 4H, *H*-3), 2.30–2.37 (m, 4H, overlapped, H_{eq}-6, H_{eq}-9), 2.01 (s, 6H, *H*-15), 1.48–1.75 (m, 4H, *H*-2), 1.09 (s, 6H, *H*-14), 0.89–1.01 (m, 4H, overlapped, H_{eq}-6, H_{eq}-9), 0.45–0.51 (m, 2H, *H*-1), 0.36–0.42 (m, 2H, *H*-5); ^{13}C NMR (125 MHz, $CDCl_3$) *δ*: 170.5 (*C*-12), 162.4 (*C*-1'), 155.2 (*C*-4), 139.0 (*C*-11), 122.6 (*C*-13), 75.6 (*C*-8), 39.1 (*C*-3), 37.7 (*C*-7), 37.2 (*C*-9), 34.3 (*C*-1), 30.7 (*C*-6), 26.1 (*C*-2), 23.0 (*C*-5), 18.4 (*C*-14), 17.3 (*C*-10), 15.9 (*C*-15); H*R*-MS (ESI): *m*/*z* calcd for $C_{32}H_{43}N_4O_6$([M + H]$^+$), 579.3177; found, 579.3183.

4. ***6d****: N'-(4-((4aS,5S,5aR)-5a-methyl-3-methylene-2-oxooctahydro-2H-cyclopropa[f]benzofuran-5-yl)butan-2-ylidene)thiophene-2-carbohydrazide*

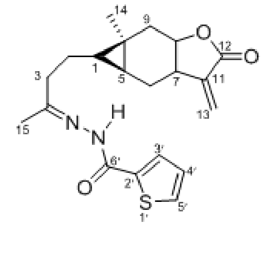

Milky-white solid, mp: 128–129 ℃ ; ^{1}H NMR (500 MHz, $CDCl_3$) δ: 10.39 (s, 1H, NH), 8.14 (d, J = 3.7 Hz, 1H, *H*-3'), 7.62 (d, J = 4.9 Hz, 1H, *H*-5'), 7.10 (dd, J = 4.9, 3.7 Hz, 1H, *H*-4'), 6.16 (d, J = 2.6 Hz, 1H, *H*-13α), 5.48 (d, J = 2.6 Hz, 1H, *H*-13β), 4.72–4.77 (m, 1H, *H*-8), 3.05–3.14 (m, 1H, *H*-7), 2.45–2.48 (t, J = 7.1 Hz, 2H, *H*-3), 2.25–2.32 (m, 2H, overlapped, H_{eq}-6, H_{eq}-9), 2.05 (s, 3H, *H*-15), 1.62–1.74 (m, 2H, *H*-2), 1.09 (s, 3H, *H*-14), 0.82–0.96 (m, 2H, overlapped, Heq-6, Heq-9), 0.48–0.54 (m, 1H, *H*-1), 0.35–0.39 (m, 1H, *H*-5); ^{13}C NMR (125 MHz, $CDCl_3$) *δ*: 170.5 (*C*-12), 163.1 (*C*-6'), 154.0 (*C*-4), 139.1 (*C*-11), 135.0 (*C*-2'), 134.5 (*C*-3'), 133.3 (*C*-5'), 126.2 (*C*-4'), 122.4 (*C*-13), 75.7 (*C*-8), 38.8 (*C*-3), 37.6 (*C*-7), 37.3 (*C*-9), 34.4 (*C*-1), 30.7 (*C*-6), 26.2 (*C*-2), 22.9 (*C*-5), 18.3 (*C*-14), 17.1 (*C*-10), 16.0 (*C*-15); H*R*-MS (ESI): *m*/*z* calcd for $C_{20}H_{25}N_2O_3S$([M + H]$^+$), 373.1580; found, 373.1581.

5. ***6e****: 5-Chloro-N'-(4-((4aS,5S,5aR)-5a-methyl-3-methylene-2-oxooctahydro-2H-cyclopropa[f]benzofuran-5-yl)butan-2-ylidene)thiophene-2-carbohydrazide*

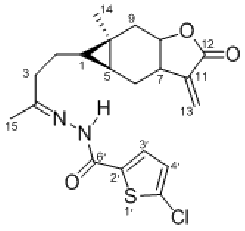

White solid, mp: 130–132 ℃ ;^{1}H NMR (500 MHz, $CDCl_3$) δ: 9.78 (s, 1H, NH), 7.90 (d, *J* = 4.2 Hz, 1H, *H*-3'), 6.96 (d, *J* = 4.2 Hz, 1H, *H*-4'), 6.23 (d, *J* = 2.6 Hz, 1H, *H*-13α), 5.53 (d, *J* = 2.6 Hz, 1H, *H*-13β), 4.76–4.81 (m, 1H, *H*-8), 3.12–3.17 (m, 1H, *H*-7), 2.50 (t, *J* = 7.1 Hz, 2H, *H*-3), 2.28–2.39 (m, 2H, overlapped, H_{eq}-6, H_{eq}-9), 2.00 (s, 3H, *H*-15), 1.49–1.81 (m, 2H, *H*-2), 1.14 (s, 3H, *H*-14), 0.89–1.00 (m, 2H, overlapped, H_{eq}-6, H_{eq}-9), 0.51–0.56 (m, 1H, *H*-1), 0.38–0.43 (m, 1H, *H*-5); ^{13}C NMR (125 MHz, $CDCl_3$) *δ*: 170.5 (*C*-12), 162.0 (*C*-6'), 153.6 (*C*-4), 139.9 (*C*-5'), 139.0 (*C*-11), 134.5 (*C*-2'), 130.2 (*C*-3'), 125.5 (*C*-4'), 122.6 (*C*-13), 75.7 (*C*-8), 38.7 (*C*-3), 37.7 (*C*-7), 37.3 (*C*-9), 34.3 (*C*-1), 30.8 (*C*-6), 26.3 (*C*-2), 23.1 (*C*-5), 18.4 (*C*-14), 17.2 (*C*-10), 15.8 (*C*-15); H*R*-MS (ESI): *m*/*z* calcd for $C_{20}H_{24}ClN_2O_3S([M + H]^+)$, 407.1191; found, 407.1191.

6. ***6f****: 5-Bromo-N'-(4-((4aS,5S,5aR)-5a-methyl-3-methylene-2-oxooctahydro-2H-cyclopropa[f]benzofuran-5-yl)butan-2-ylidene)thiophene-2-carbohydrazide*

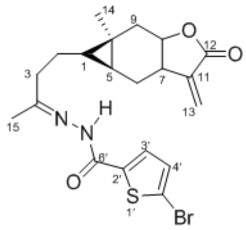

Milky-white solid, mp: 118–119 ℃ ; ^{1}H NMR (500 MHz, $CDCl_3$) δ: 9.86 (s, 1H, NH), 7.86 (d, *J* = 4.2 Hz, 1H, *H*-3'), 7.09 (d, *J* = 4.2 Hz, 1H, *H*-4'), 6.22 (d, *J* = 2.5 Hz, 1H, *H*-13α), 5.53 (d, *J* = 2.5 Hz, 1H, *H*-13β), 4.76–4.81 (m, 1H, *H*-8), 3.12–3.18 (m, 1H, *H*-7), 2.49 (t, *J* = 7.1 Hz, 2H, *H*-3), 2.28–2.39 (m, 2H, overlapped, H_{eq}-6, H_{eq}-9), 2.01 (s, 3H, *H*-15), 1.57–1.80 (m, 2H, *H*-2), 1.15 (s, 3H, *H*-14), 0.89–1.00 (m, 2H, overlapped, H_{eq}-6, H_{eq}-9), 0.51–0.56 (m, 1H, *H*-1), 0.38–0.44 (m, 1H, *H*-5); ^{13}C NMR (125 MHz, $CDCl_3$) *δ*: 170.5 (*C*-12), 162.0 (*C*-6'), 153.8 (*C*-4), 139.0 (*C*-11), 135.1 (*C*-5'), 133.2 (*C*-2'), 129.1 (*C*-3'), 123.6 (*C*-4'), 122.6 (*C*-13), 75.7 (*C*-8), 38.7 (*C*-3), 37.8 (*C*-7), 37.3 (*C*-9), 34.3 (*C*-1), 30.8 (*C*-6), 26.3 (*C*-2), 23.1 (*C*-5), 18.4 (*C*-14), 17.2 (*C*-10), 15.9 (*C*-15); H*R*-MS (ESI): *m*/*z* calcd for $C_{20}H_{24}BrN_2O_3S([M + H]^+)$, 451.0686; found, 451.0684.

*7. **6g**: N'-(4-((4aS,5S,5aR)-5a-methyl-3-methylene-2-oxooctahydro-2H-cyclopropa[f]benzofuran-5-yl) butan-2-ylidene)nicotinohydrazide*

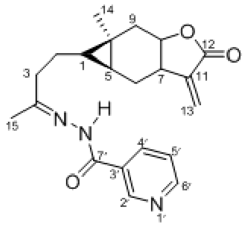

Colourless solid, mp: 46–47 ℃ ; ^{1}H NMR (500 MHz, $CDCl_3$) δ: 9.79 (s, 1H, NH), 9.02 (s, 1H, *H*-2’), 8.65 (d, *J* = 6.7 Hz, 1H, *H*-6’), 8.16 (d, *J* = 7.5 Hz, 1H, *H*-4’), 7.34 (dd, *J* = 7.5, 6.7 Hz, 1H, *H*-5’), 6.16 (d, *J* = 2.6 Hz, 1H, *H*-13α), 5.54 (d, *J* = 2.6 Hz, 1H, *H*-13β), 4.74–4.79 (m, 1H, *H*-8), 3.10–3.17 (m, 1H, *H*-7), 2.42 (t, *J* = 7.6 Hz, 2H, *H*-3), 2.24–2.29 (m, 2H, overlapped, H_{eq}-6, H_{eq}-9), 2.04 (s, 3H, *H*-15), 1.41–1.68 (m, 2H, *H*-2), 1.08 (s, 3H, *H*-14), 0.85–0.98 (m, 2H, overlapped, H_{eq}-6, H_{eq}-9), 0.31–0.49 (m, 2H, *H*-1, *H*-5); ^{13}C NMR (125 MHz, $CDCl_3$) *δ*: 170.6 (*C*-12), 161.6 (*C*-7’), 152.0 (*C*-4), 148.4 (*C*-2’), 148.3 (*C*-6’), 139.0 (*C*-11), 135.5 (*C*-4’), 129.7 (*C*-3’), 123.3 (*C*-5’), 122.5 (*C*-13), 75.7 (*C*-8), 39.0 (*C*-3), 37.4 (*C*-7), 37.0 (*C*-9), 34.1 (*C*-1), 30.5 (*C*-6), 26.2 (*C*-2), 22.8 (*C*-5), 18.1 (*C*-14), 17.0 (*C*-10), 14.1 (*C*-15); H*R*-MS (ESI): *m*/*z* calcd for $C_{21}H_{26}N_3O_3$([M + H]$^{+}$), 368.1969; found, 368.1969.

*8. **6h**: 4-Amino-N'-(4-((4aS,5S,5aR)-5a-methyl-3-methylene-2-oxooctahydro-2H-cyclopropa[f]benzofuran -5-yl)butan-2-ylidene)benzohydrazide*

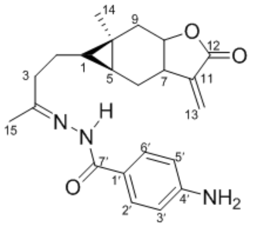

White solid, mp: 75–77 ℃ ; ^{1}H NMR (500 MHz, $CDCl_3$) δ: 9.04 (s, 1H, NH), 7.62 (d, *J* = 7.9 Hz, 2H, *H*-3’, *H*-5’), 6.60 (d, *J* = 7.9 Hz, 2H, *H*-2’, *H*-6’), 6.14 (d, *J* = 2.6 Hz, 1H, *H*-13α), 5.54 (d, *J* = 2.6 Hz, 1H, *H*-13β), 4.66–4.77 (m, 1H, *H*-8), 4.29 (s, 2H, NH_2), 3.07–3.11 (m, 1H, *H*-7), 2.42 (t, *J* = 7.6 Hz, 2H, *H*-3), 2.25–2.29 (m, 2H, overlapped, H_{eq}-6, H_{eq}-9), 1.95 (s, 3H, *H*-15), 1.41–1.66 (m, 2H, *H*-2), 1.03 (s, 3H, *H*-14), 0.79–0.92 (m, 2H, overlapped, H_{eq}-6, H_{eq}-9), 0.40–0.45 (m, 1H, *H*-1), 0.29–0.35 (m, 1H, *H*-5); ^{13}C NMR (125 MHz, $CDCl_3$) *δ*: 170.7 (*C*-12), 164.2 (*C*-7’), 158.0 (*C*-4), 150.8 (*C*-4’), 139.0 (*C*-11), 129.2 (*C*-2’, 6’), 122.6 (*C*-13), 121.7 (*C*-1’), 113.8 (*C*-3’, *C*-5’), 75.8 (*C*-8), 38.9 (*C*-3), 37.4 (*C*-7), 37.0 (*C*-9), 34.1 (*C*-1), 30.5 (*C*-6), 26.2 (*C*-2), 22.8 (*C*-5), 18.2 (*C*-14), 17.0 (*C*-10), 15.5 (*C*-15); H*R*-MS (ESI): *m*/*z* calcd for $C_{22}H_{28}N_3O_3$([M + H]$^{+}$), 382.2125; found, 382.2124.

9. ***6i****: 4-Hydroxy-N'-(4-((4aS,5S,5aR)-5a-methyl-3-methylene-2-oxooctahydro-2H-cyclopropa[f] benzofuran-5-yl)butan-2-ylidene)benzohydrazide*

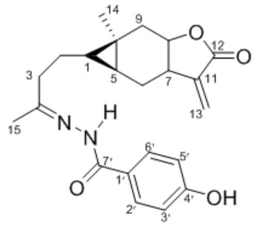

White solid, mp: 63–64 ℃ ; ^{1}H NMR (500 MHz, $CDCl_3$) δ: 9.24 (s, 1H, NH), 7.60 (d, *J* = 7.3 Hz, 2H, *H*-2’, *H*-6’), 6.88 (d, *J* = 7.3 Hz, 2H, *H*-3’, *H*-5’), 6.23 (d, *J* = 2.6 Hz, 1H, *H*-13α), 5.57 (d, *J* = 2.6 Hz, 1H, *H*-13β), 4.66–4.74 (m, 1H, *H*-8), 3.03–3.11 (m, 1H, *H*-7), 2.46 (t, *J* = 7.6 Hz, 2H, *H*-3), 2.25–2.30 (m, 2H, overlapped, H_{eq}-6, H_{eq}-9), 2.16 (s, 3H, *H*-15), 1.59–1.62 (m, 2H, *H*-2), 0.99 (s, 3H, *H*-14), 0.79–0.94 (m, 2H, overlapped, H_{eq}-6, H_{eq}-9), 0.29–0.45 (m, 2H, *H*-1, *H*-5); ^{13}C NMR (125 MHz, $CDCl_3$) *δ*: 171.0 (*C*-12), 165.0 (*C*-7’), 161.0 (*C*-4’), 155.1 (*C*-4), 139.0 (*C*-11), 129.3 (*C*-2’, 6’), 123.8 (*C*-1’), 122.7 (*C*-13), 115.8 (*C*-3’, *C*-5’), 75.8 (*C*-8), 38.8 (*C*-3), 37.7 (*C*-7), 37.3 (*C*-9), 34.2 (*C*-1), 30.7 (*C*-6), 26.0 (*C*-2), 22.9 (*C*-5), 18.2 (*C*-14), 17.0 (*C*-10), 15.9 (*C*-15); H*R*-MS (ESI): *m*/*z* calcd for $C_{22}H_{27}N_2O_4$([M + H]$^+$), 383.1965; found, 383.1963.

10. ***6j****: 2-Hydroxy-N'-(4-((4aS,5S,5aR)-5a-methyl-3-methylene-2-oxooctahydro-2H-cyclopropa[f] benzofuran-5-yl)butan-2-ylidene)benzohydrazide*

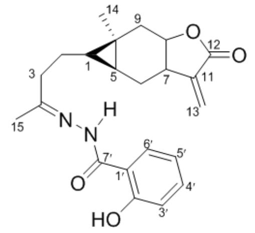

White solid, mp: 70–71 ℃ ; ^{1}H NMR (500 MHz, $CDCl_3$) δ: 6.86–7.62 (m, 4H, *H*-3’, *H*-4’, *H*-5’, *H*-6’), 6.21 (d, *J* = 2.6 Hz, 1H, *H*-13α), 5.55 (d, *J* = 2.6 Hz, 1H, *H*-13β), 4.74–4.78 (m, 1H, *H*-8), 3.13–3.15 (m, 1H, *H*-7), 2.46 (t, *J* = 7.5 Hz, 2H, *H*-3), 2.23–2.38 (m, 2H, overlapped, H_{eq}-6, H_{eq}-9), 2.01 (s, 3H, *H*-15), 1.52–1.74 (m, 2H, *H*-2), 1.07 (s, 3H, *H*-14), 0.84–0.99 (m, 2H, overlapped, H_{eq}-6, H_{eq}-9), 0.35–0.45 (m, 2H, *H*-1, *H*-5); ^{13}C NMR (125 MHz, $CDCl_3$) *δ*: 170.7 (*C*-12), 166.1 (*C*-7’), 161.1 (*C*-2’), 160.3 (*C*-4), 139.2 (*C*-11), 134.4 (*C*-1’), 126.3 (*C*-4’), 122.7 (*C*-13), 119.0 (*C*-6’), 118.5 (*C*-5’), 114.2 (*C*-3’), 75.8 (*C*-8), 38.9 (*C*-3), 37.7 (*C*-7), 37.3 (*C*-9), 34.3 (*C*-1), 30.5 (*C*-6), 26.1 (*C*-2), 22.9 (*C*-5), 18.4 (*C*-14), 17.1 (*C*-10), 16.0 (*C*-15); H*R*-MS (ESI): *m*/*z* calcd for $C_{22}H_{27}N_2O_4$([M + H]$^+$), 383.1965; found, 383.1964.

*11. **6k**: 2-Chloro-N'-(4-((4aS,5S,5aR)-5a-methyl-3-methylene-2-oxooctahydro-2H-cyclopropa[f] benzofuran-5-yl)butan-2-ylidene)benzohydrazide*

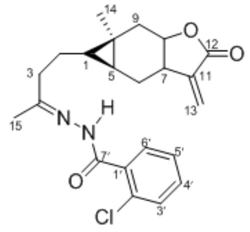

Light yellow solid, mp: 60–61 ℃ ; ^{1}H NMR (500 MHz, $CDCl_3$) δ: 9.33 (s, 1H, NH), 7.25–7.63 (m, 4H, *H*-3',*H*-4', *H*-5', *H*-6'), 6.14 (d, *J* = 2.6 Hz, 1H, *H*-13α), 5.53 (d, *J* = 2.6 Hz, 1H, *H*-13β), 4.68–4.78 (m, 1H, *H*-8), 3.09–3.15 (m, 1H, *H*-7), 2.46 (t, *J* = 7.6 Hz, 2H, *H*-3), 2.25–2.31 (m, 2H, overlapped, H_{eq}-6, H_{eq}-9),1.96 (s, 3H, *H*-15), 1.21–1.74 (m, 2H, *H*-2), 1.09 (s, 3H, *H*-14), 0.74–0.98 (m, 2H, overlapped, H_{eq}-6, H_{eq}-9), 0.20–0.48 (m, 2H, *H*-1, *H*-5); ^{13}C NMR (125 MHz, $CDCl_3$) *δ*: 170.4 (*C*-12), 162.6 (*C*-7'), 159.9 (*C*-2'), 152.4 (*C*-4), 139.0 (*C*-11), 131.4 (*C*-1'), 130.8 (*C*-5'), 130.4 (*C*-6'), 130.0 (*C*-3'), 127.0 (*C*-4'), 122.4 (*C*-13), 75.7 (*C*-8), 39.0 (*C*-3), 37.5 (*C*-7), 37.1 (*C*-9), 34.2 (*C*-1), 30.6 (*C*-6), 26.1 (*C*-2), 22.9 (*C*-5), 18.3 (*C*-14), 17.1 (*C*-10), 16.1 (*C*-15); H*R*-MS (ESI): *m*/*z* calcd for $C_{22}H_{26}ClN_2O_3$([M + H]$^+$), 401.1627; found, 401.1626.

*12. **6l**: 3-Chloro-N'-(4-((4aS,5S,5aR)-5a-methyl-3-methylene-2-oxooctahydro-2H-cyclopropa[f] benzofuran-5-yl)butan-2-ylidene)benzohydrazide*

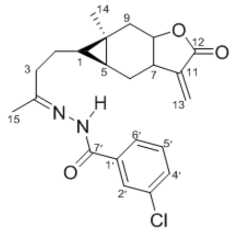

Light yellow solid, mp: 66–67 ℃ ; ^{1}H NMR (500 MHz, $CDCl_3$) δ: 9.09 (s, 1H, NH), 7.80 (d, *J* = 7.4 Hz, 1H, *H*-4'), 7.69 (s, 1H, *H*-2'), 7.46 (d, *J* = 7.7 Hz, 1H, *H*-6'), 7.34 (dd, *J* = 7.7, 7.4 Hz, 1H, *H*-5'), 6.18 (d, *J* = 2.5 Hz, 1H, *H*-13α), 5.54 (d, *J* = 2.5 Hz, 1H, *H*-13β), 4.73–4.78 (m, 1H, *H*-8), 3.12–3.16 (m, 1H, *H*-7), 2.47 (t, *J* = 7.6 Hz, 2H, *H*-3), 2.27–2.33 (m, 2H, overlapped, H_{eq}-6, H_{eq}-9), 2.11 (s, 3H, *H*-15), 1.47–1.68 (m, 2H, *H*-2), 1.07 (s, 3H, *H*-14), 0.89–0.99 (m, 2H, overlapped, H_{eq}-6, H_{eq}-9), 0.38–0.45 (m, 2H, *H*-1, *H*-5); ^{13}C NMR (125 MHz, $CDCl_3$) *δ*: 170.5 (*C*-12), 162.9 (*C*-7'), 160.5 (*C*-4), 139.1 (*C*-11), 135.6 (*C*-3'), 134.7 (*C*-4'), 131.6 (*C*-1'), 129.9 (*C*-6'), 127.6 (*C*-5'), 125.5 (*C*-2'), 122.5 (*C*-13), 75.7 (*C*-8), 39.0 (*C*-3), 37.7 (*C*-7), 37.1 (*C*-9), 34.2 (*C*-1), 30.6 (*C*-6), 26.2 (*C*-2), 22.9 (*C*-5), 18.2 (*C*-14), 17.1 (*C*-10), 16.0 (*C*-15); H*R*-MS (ESI): *m*/*z* calcd for $C_{22}H_{26}ClN_2O_3$([M + H]$^+$), 401.1627; found, 401.1626.

13. ***6m****: N'-(4-((4aS,5S,5aR)-5a-methyl-3-methylene-2-oxooctahydro-2H-cyclopropa[f]benzofuran-5-yl)butan-2-ylidene)-4-nitrobenzohydrazide*

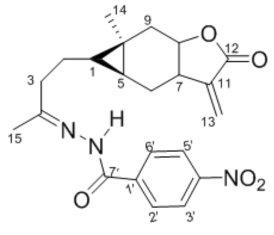

Yellow solid, mp: 69–70 ℃ ; ^{1}H NMR (500 MHz, $CDCl_3$) δ: 9.21 (s, 1H, NH), 7.80 (d, *J* = 7.7 Hz, 1H, *H*-3'), 7.69 (d, *J* = 7.7 Hz, 1H, *H*-2'), 7.43 (d, *J* = 7.4 Hz, 1H, *H*-5'), 7.35 (d, *J* = 7.4 Hz, 1H, *H*-6'), 6.17 (d, *J* = 2.4 Hz, 1H, *H*-13α), 5.54 (d, *J* = 2.4 Hz, 1H, *H*-13β), 4.72–4.76 (m, 1H, *H*-8), 3.13–3.14 (m, 1H, *H*-7), 2.46 (t, *J* = 7.6 Hz, 2H, *H*-3), 2.24–2.31 (m, 2H, overlapped, H_{eq}-6, H_{eq}-9), 2.01 (s, 3H, *H*-15), 1.48–1.67 (m, 2H, *H*-2), 1.07 (s, 3H, *H*-14), 0.84–0.99 (m, 2H, overlapped, H_{eq}-6, H_{eq}-9), 0.42–0.45 (m, 1H, *H*-1), 0.29–0.40 (m, 1H, *H*-5); ^{13}C NMR (125 MHz, $CDCl_3$) *δ*: 170.5 (*C*-12), 163.0 (*C*-7'), 160.7 (*C*-4), 145.6 (*C*-4'), 139.1 (*C*-11), 134.5 (*C*-1'), 130.5 (*C*-6'), 129.8 (*C*-2'), 125.7 (*C*-3'), 125.5 (*C*-5'), 122.5 (*C*-13), 75.7 (*C*-8), 39.0 (*C*-3), 37.6 (*C*-7), 37.1 (*C*-9), 34.2 (*C*-1), 30.5 (*C*-6), 26.2 (*C*-2), 22.9 (*C*-5), 18.2 (*C*-14), 17.1 (*C*-10), 16.1 (*C*-15); H*R*-MS (ESI): *m*/*z* calcd for $C_{22}H_{26}N_3O_5$([M + H]$^+$), 412.1867; found, 412.1866.

14. ***6n****: 4-Cyano-N'-(4-((4aS,5S,5aR)-5a-methyl-3-methylene-2-oxooctahydro-2H-cyclopropa[f] benzofuran-5-yl)butan-2-ylidene)benzohydrazide*

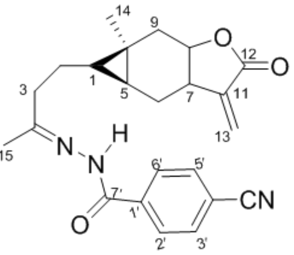

White solid, mp: 87–88 ℃ ; ^{1}H NMR (500 MHz, $CDCl_3$) δ: 9.38 (s, 1H, NH), 7.96 (d, *J* = 7.7 Hz, 2H, *H*-3', *H*-5'), 7.76 (d, *J* = 7.7 Hz, 2H, *H*-2', *H*-6'), 6.18 (d, *J* = 2.6 Hz, 1H, *H*-13α), 5.55 (d, *J* = 2.6 Hz, 1H, *H*-13β), 4.74–4.79 (m, 1H, *H*-8), 3.15–3.16 (m, 1H, *H*-7), 2.46 (t, *J* = 7.6 Hz, 2H, *H*-3), 2.26–2.33 (m, 2H, overlapped, H_{eq}-6, H_{eq}-9), 1.52–1.55 (m, 2H, *H*-2), 1.08 (s, 3H, *H*-14), 0.84–0.99 (m, 2H, overlapped, H_{eq}-6, H_{eq}-9), 0.41–0.46 (m, 2H, *H*-1, *H*-5); ^{13}C NMR (125 MHz, $CDCl_3$) *δ*: 170.6 (*C*-12), 161.9 (*C*-7'), 154.4 (*C*-4), 139.0 (*C*-11), 137.8 (*C*-1'), 132.3 (*C*-2', *C*-6'), 128.2 (*C*-3', *C*-5'), 122.6 (*C*-13), 118.0 (*C*-8'), 115.0 (*C*-4'), 75.7 (*C*-8), 39.0 (*C*-3), 37.7 (*C*-7), 37.2 (*C*-9), 34.2 (*C*-1), 30.7 (*C*-6), 26.2 (*C*-2), 22.9 (*C*-5), 18.2 (*C*-14), 17.1 (*C*-10), 16.2 (*C*-15); H*R*-MS (ESI): *m*/*z* calcd for $C_{23}H_{26}N_3O_3$([M + H]$^+$), 392.1969; found, 392.1965.

*15. **6o**: 3-Methyl-N'-(4-((4aS,5S,5aR)-5a-methyl-3-methylene-2-oxooctahydro-2H-cyclopropa[f] benzofuran-5-yl)butan-2-ylidene)benzohydrazide*

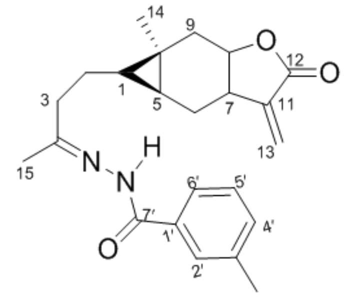

Milky-white solid, mp: 66–67 ℃ ; ^{1}H NMR (500 MHz, $CDCl_3$) δ: 9.16 (s, 1H, NH), 7.63 (d, *J* = 7.9 Hz, 1H, *H*-6'), 7.61 (dd, *J* = 8.1, 7.9 Hz, 1H, *H*-5'), 7.38 (s, 1H, *H*-2'), 7.28 (d, *J* = 8.1 Hz, 1H, *H*-4'), 6.16 (d, *J* = 2.6 Hz, 1H, *H*-13α), 5.53 (d, *J* = 2.6 Hz, 1H, *H*-13β), 4.71–4.76 (m, 1H, *H*-8), 3.10–3.14 (m, 1H, *H*-7), 2.44 (t, *J* = 7.6 Hz, 2H, *H*-3), 2.37 (s, 3H, Me), 2.26–2.31 (m, 2H, overlapped, H_{eq}-6, H_{eq}-9), 2.01 (s, 3H, *H*-15), 1.46–1.61 (m, 2H, *H*-2), 1.07 (s, 3H, *H*-14), 0.89–0.95 (m, 2H, overlapped, H_{eq}-6, H_{eq}-9), 0.37–0.45 (m, 2H, *H*-1, *H*-5); ^{13}C NMR (125 MHz, $CDCl_3$) *δ*: 170.5 (*C*-12), 164.2 (*C*-7'), 159.3 (*C*-4), 139.1 (*C*-11), 138.3 (*C*-3'), 133.7 (*C*-1'), 132.3 (*C*-5'), 128.3 (*C*-6'), 128.0 (*C*-4'), 124.2 (*C*-2'), 122.4 (*C*-13), 75.7 (*C*-8), 39.0 (*C*-3), 37.5 (*C*-7), 37.1 (*C*-9), 34.1 (*C*-1), 30.5 (*C*-6), 26.2 (*C*-2), 22.9 (*C*-5), 21.2 (*C*-8'), 18.3 (*C*-14), 17.0 (*C*-10), 15.8 (*C*-15); H*R*-MS (ESI): *m*/*z* calcd for $C_{23}H_{29}N_2O_3$([M + H]$^+$), 381.2173; found, 381.2172.

*16. **6p**: 4-Methoxy-N'-(4-((4aS,5S,5aR)-5a-methyl-3-methylene-2-oxooctahydro-2H-cyclopropa[f] benzofuran-5-yl)butan-2-ylidene)benzohydrazide*

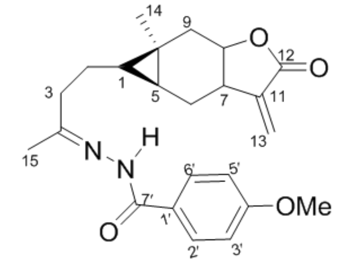

White solid, mp: 69–71 ℃ ; ^{1}H NMR (500 MHz, $CDCl_3$) δ: 8.78 (s, 1H, NH), 7.81 (d, *J* = 7.7 Hz, 2H, *H*-3', *H*-5'), 6.93 (d, *J* = 7.7 Hz, 2H, *H*-2', *H*-6'), 6.22 (d, *J* = 2.6 Hz, 1H, *H*-13α), 5.54 (d, *J* = 2.6 Hz, 1H, *H*-13β), 4.74–4.79 (m, 1H, *H*-8), 3.85 (s, 3H, -OCH_3), 3.12–3.18 (m, 1H, *H*-7), 2.48 (t, *J* = 7.6 Hz, 2H, *H*-3), 2.28–2.35 (m, 2H, overlapped, H_{eq}-6, H_{eq}-9), 2.16 (s, 3H, *H*-15), 1.52–1.61 (m, 2H, *H*-2), 1.09 (s, 3H, *H*-14), 0.92–0.97 (m, 2H, overlapped, H_{eq}-6, H_{eq}-9), 0.37–0.47 (m, 2H, *H*-1, *H*-5); ^{13}C NMR (125 MHz, $CDCl_3$) *δ*: 170.4 (*C*-12), 162.4 (*C*-7'), 160.2 (*C*-4'), 156.4 (*C*-4), 139.0 (*C*-11), 129.2 (*C*-2', *C*-6'), 125.8 (*C*-1'), 122.5 (*C*-13), 113.9 (*C*-3', *C*-5'), 75.6 (*C*-8), 55.4 (*C*-8'), 39.0 (*C*-3), 37.7 (*C*-7), 37.3 (*C*-9), 34.2 (*C*-1), 30.7 (*C*-6), 26.2 (*C*-2), 22.9 (*C*-5), 18.2 (*C*-14), 17.2 (*C*-10), 15.4 (*C*-15); H*R*-MS (ESI): *m*/*z* calcd for $C_{23}H_{29}N_2O_4$([M + H]$^+$), 397.2122; found, 397.2121.

17. ***6q****: 3-Methoxy-N'-(4-((4aS,5S,5aR)-5a-methyl-3-methylene-2-oxooctahydro-2H-cyclopropa[f] benzofuran-5-yl)butan-2-ylidene)benzohydrazide*

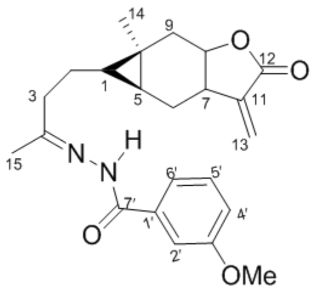

White solid, mp: 54–55 ℃ ; ^{1}H NMR (500 MHz, $CDCl_3$) δ: 9.03 (s, 1H, NH), 7.29–7.37 (m, 3H, *H*-4', *H*-5', *H*-6'), 7.03 (s, 1H, *H*-2'), 6.18 (d, *J* = 2.6 Hz, 1H, *H*-13α), 5.54 (d, *J* = 2.6 Hz, 1H, *H*-13β), 4.73–4.78 (m, 1H, *H*-8), 3.82 (s, 3H, -OCH_3), 3.13–3.15 (m, 1H, *H*-7), 2.48 (t, *J* = 7.6 Hz, 2H, *H*-3), 2.26–2.31 (m, 2H, overlapped, H_{eq}-6, H_{eq}-9), 2.14 (s, 3H, *H*-15), 1.51–1.61 (m, 2H, *H*-2), 1.07 (s, 3H, *H*-14), 0.89–0.96 (m, 2H, overlapped, H_{eq}-6, H_{eq}-9), 0.38–0.46 (m, 2H, *H*-1, *H*-5); ^{13}C NMR (125 MHz, $CDCl_3$) *δ*: 170.5 (*C*-12), 163.8 (*C*-7'), 159.7 (*C*-3'), 153.6 (*C*-4), 139.0 (*C*-11), 135.1 (*C*-1'), 129.6 (*C*-5'), 122.5 (*C*-13), 119.0 (*C*-6'), 117.7 (*C*-2'), 112.8 (*C*-4'), 75.7 (*C*-8), 55.4 (*C*-8'), 39.0 (*C*-3), 37.6 (*C*-7), 37.1 (*C*-9), 34.2 (*C*-1), 30.6 (*C*-6), 26.2 (*C*-2), 22.9 (*C*-5), 18.2 (*C*-14), 17.1 (*C*-10), 15.8 (*C*-15); H*R*-MS (ESI): *m*/*z* calcd for $C_{23}H_{29}N_2O_4$([M + H]$^+$), 397.2122; found, 397.2121.

18. ***7r****: N'-((E)-4-((4aS,5S,5aR)-5a-methyl-3-methylene-2-oxooctahydro-2H-cyclopropa[f]benzofuran-5-yl)butan-2-ylidene)benzenesulfonohydrazide*

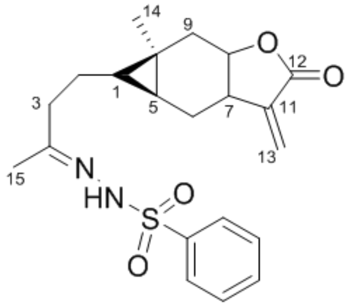

Light yellow solid, mp: 49–50 ℃ ; ^{1}H NMR (500 MHz, $CDCl_3$) δ: 7.96 (d, *J* = 8.7 Hz, 2H, *H*-2', *H*-6'), 7.45–7.58 (m, 3H, *H*-3', *H*-4', *H*-5'), 6.22 (d, *J* = 2.5 Hz, 1H, *H*-13α), 5.56 (d, *J* = 2.5 Hz, 1H, *H*-13β), 4.70–4.75 (m, 1H, *H*-8), 3.07–3.13 (m, 1H, *H*-7), 2.48 (t, *J* = 7.6 Hz, 2H, *H*-3), 2.26–2.31 (m, 2H, overlapped, H_{eq}-6, H_{eq}-9), 1.22–1.37 (m, 2H, *H*-2), 0.99 (s, 3H, *H*-14), 0.78–0.91 (m, 2H, overlapped, H_{eq}-6, H_{eq}-9), 0.21–0.39 (m, 2H, *H*-1, *H*-5); ^{13}C NMR (125 MHz, $CDCl_3$) *δ*: 170.8 (*C*-12), 158.8 (*C*-4), 139.0 (*C*-11), 138.4 (*C*-1'), 133.1 (*C*-4'), 128.8 (*C*-3', 5'), 128.0 (*C*-2', *C*-6'), 122.7 (*C*-13), 75.7 (*C*-8), 38.5 (*C*-3), 37.6 (*C*-7), 37.1 (*C*-9), 33.8 (*C*-1), 30.5 (*C*-6), 25.4 (*C*-2), 22.9 (*C*-5), 18.2 (*C*-14), 16.8 (*C*-10), 16.1 (*C*-15); H*R*-MS (ESI): *m*/*z* calcd for $C_{21}H_{27}N_2O_4S$([M + H]$^+$), 403.1686; found, 403.1689.

19. ***7s****: 4-Methyl-N'-((E)-4-((4aS,5S,5aR)-5a-methyl-3-methylene-2-oxooctahydro-2H-cyclopropa[f] benzofuran-5-yl)butan-2-ylidene)benzenesulfonohydrazide*

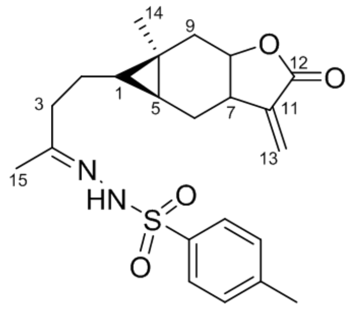

Colourless solid, mp: 55–56 ℃ ; ^{1}H NMR (500 MHz, $CDCl_3$) δ: 7.74 (d, *J* = 7.9 Hz, 2H, *H*-2', *H*-6'), 7.29 (d, *J* = 7.9 Hz, 2H, *H*-3', *H*-5'), 6.24 (d, *J* = 2.6 Hz, 1H, *H*-13α), 5.56 (d, *J* = 2.6 Hz, 1H, *H*-13β), 4.71–4.75 (m, 1H, *H*-8), 3.09–3.18 (m, 1H, *H*-7), 2.40 (t, *J* = 7.6 Hz, 2H, *H*-3), 2.24–2.30 (m, 2H, overlapped, H_{eq}-6, H_{eq}-9), 1.79 (s, 3H, *H*-15), 1.51–1.65 (m, 2H, *H*-2), 1.26 (s, 3H, *H*-7'), 1.02 (s, 3H, *H*-14), 0.81–0.97 (m, 2H, overlapped, H_{eq}-6, H_{eq}-9), 0.28–0.47 (m, 2H, *H*-1, *H*-5); ^{13}C NMR (125 MHz, $CDCl_3$) *δ*: 170.5 (*C*-12), 158.1 (*C*-4), 144.0 (*C*-4'), 139.1 (*C*-11), 135.5 (*C*-1'), 129.4 (*C*-3', 5'), 128.1 (*C*-2', *C*-6'), 122.5 (*C*-13), 75.6 (*C*-8), 38.5 (*C*-3), 37.7 (*C*-7), 37.2 (*C*-9), 34.0 (*C*-1), 30.6 (*C*-6), 25.5 (*C*-2), 23.0 (*C*-5), 21.5 (*C*-7'), 18.2 (*C*-14), 17.0 (*C*-10), 16.0 (*C*-15); H*R*-MS (ESI): *m*/*z* calcd for $C_{22}H_{29}N_2O_4S([M + H]^+)$, 417.1843; found, 417.1845.

20. ***8a****: (4aS,5S,5aR)-5-(3-(2-(2-hydroxyethyl)hydrazono)butyl)-5a-methyl-3-methyleneoctahydro-2H-cyclopropa[f]benzofuran-2-one*

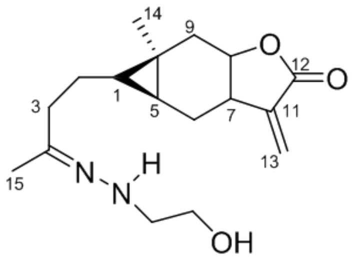

Milky-white ropy liquid; ^{1}H NMR (500 MHz, $CDCl_3$) δ: 6.24 (d, *J* = 2.6 Hz, 1H, *H*-13α), 5.5(d, *J* = 2.6 Hz, 1H, *H*-13β), 4.74–4.81 (m, 1H, *H*-8), 3.82 (t, *J* = 4.6 Hz, 2H, *H*-2'), 3.72 (s, 1H, -OH), 3.27 (t, *J* = 4.6 Hz, 2H, *H*-1'), 3.13–3.19 (m, 1H, *H*-7), 2.40 (t, *J* = 7.6 Hz, 2H, *H*-3), 2.24–2.30 (m, 2H, overlapped, H_{eq}-6, H_{eq}-9), 2.17 (s, 3H, *H*-15), 1.48–1.63 (m, 2H, *H*-2), 1.09 (s, 3H, *H*-14), 0.86–0.98 (m, 2H, overlapped, H_{eq}-6, H_{eq}-9), 0.28–0.46 (m, 2H, *H*-1, *H*-5); ^{13}C NMR (125 MHz, $CDCl_3$) *δ*: 170.4 (*C*-12), 150.0 (*C*-4), 139.1 (*C*-11), 122.5 (*C*-13), 75.6 (*C*-8), 63.7 (*C*-2'), 51.9 (*C*-1'), 38.8 (*C*-3), 37.7 (*C*-7), 37.3 (*C*-9), 34.3 (*C*-1), 30.7 (*C*-6), 23.4 (*C*-2), 23.0 (*C*-5), 18.2 (*C*-14), 17.2 (*C*-10), 14.5 (*C*-15); H*R*-MS (ESI): *m*/*z* calcd for $C_{17}H_{27}N_2O_3([M + H]^+)$, 307.1940; found, 307.1938.

21. ***8b****: (4aS,5S,5aR)-5a-methyl-3-methylene-5-(3-(2-phenylhydrazono)butyl)octahydro-2H-cyclopropa[f]benzofuran-2-one*

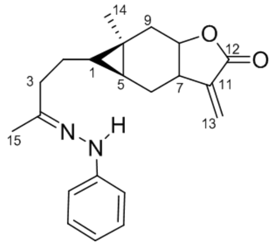

Yellow solid, mp: 41–42 ℃ ; ^{1}H NMR (500 MHz, $CDCl_3$) δ: 7.35–7.47 (m, 6H, Ar-H, –NH), 6.22 (d, *J* = 2.5 Hz, 1H, *H*-13α), 5.54 (d, *J* = 2.5 Hz, 1H, *H*-13β), 4.75–4.77 (m, 1H, *H*-8), 3.09–3.17 (m, 1H, *H*-7), 2.44 (t, *J* = 7.6 Hz, 2H, *H*-3), 2.25–2.31 (m, 2H, overlapped, H_{eq}-6, H_{eq}-9), 1.55 (s, 3H, *H*-15), 1.22–1.45 (m, 2H, *H*-2), 1.06 (s, 3H, *H*-14), 0.88–0.96 (m, 2H, overlapped, H_{eq}-6, H_{eq}-9), 0.33–0.47 (m, 2H, *H*-1, *H*-5); ^{13}C NMR (125 MHz, $CDCl_3$) *δ*: 170.7 (*C*-12), 151.0 (*C*-4), 139.1 (*C*-11), 131.4 (*C*-1’), 129.2 (*C*-3’, *C*-5’), 122.6 (*C*-13), 122.5 (*C*-2’, *C*-6’), 104.9 (*C*-4’), 75.8 (*C*-8), 38.5 (*C*-3), 37.7 (*C*-7), 37.3 (*C*-9), 34.2 (*C*-1), 30.8 (*C*-6), 26.3 (*C*-2), 22.9 (*C*-5), 18.2 (*C*-14), 17.2 (*C*-10), 15.7 (*C*-15); H*R*-MS (ESI): *m*/*z* calcd for $C_{21}H_{27}N_2O_2$([M + H]$^+$), 339.2067; found, 339.2068.

22. ***8c****: (4aS,5S,5aR)-5a-methyl-3-methylene-5-(3-(2-(2,4,6-trichlorophenyl)hydrazono)butyl) octahydro-2H-cyclopropa[f]benzofuran-2-one*

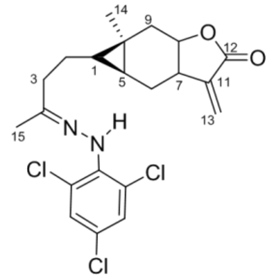

Light yellow solid, mp: 72–73 ℃ ; ^{1}H NMR (500 MHz, $CDCl_3$) δ: 9.21 (s, 1H, NH), 7.42 (s, 2H, *H*-3’, *H*-5’), 6.23 (d, *J* = 2.6 Hz, 1H, *H*-13α), 5.55 (d, *J* = 2.6 Hz, 1H, *H*-13β), 4.76–4.81 (m, 1H, *H*-8), 3.13–3.19 (m, 1H, *H*-7), 2.46 (t, *J* = 7.6 Hz, 2H, *H*-3), 2.25–2.31 (m, 2H, overlapped, H_{eq}-6, H_{eq}-9), 1.62 (s, 3H, *H*-15), 1.41–1.68 (m, 2H, *H*-2), 1.10 (s, 3H, *H*-14), 0.87–1.00 (m, 2H, overlapped, H_{eq}-6, H_{eq}-9), 0.36–0.52 (m, 2H, *H*-1, *H*-5); ^{13}C NMR (125 MHz, $CDCl_3$) *δ*: 170.6 (*C*-12), 153.3 (*C*-4), 145.5 (*C*-1’), 139.1 (*C*-11), 128.7 (*C*-2’, *C*-3’, *C*-5’, *C*-6’), 127.0 (*C*-4’), 122.5 (*C*-13), 75.7 (*C*-8), 38.6 (*C*-3), 37.8 (*C*-7), 37.4 (*C*-9), 34.5 (*C*-1), 30.8 (*C*-6), 26.1 (*C*-2), 23.0 (*C*-5), 18.3 (*C*-14), 17.2 (*C*-10), 15.1 (*C*-15); H*R*-MS (ESI): *m*/*z* calcd for $C_{21}H_{24}Cl_3N_2O_2$([M + H]$^+$), 441.0898; found, 441.0896.

23. ***8d****: (4aS,5S,5aR)-5a-methyl-3-methylene-5-(3-(2-(4-nitrophenyl)hydrazono)butyl)octahydro-2H-cyclopropa[f]benzofuran-2-one*

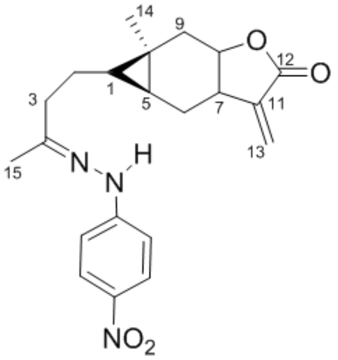

Yellow solid, mp: 93–94 ℃ ; ^{1}H NMR (500 MHz, $CDCl_3$) δ: 8.12 (d, *J* = 8.4 Hz, 2H, *H*-3', *H*-5'), 7.74 (s, 1H, NH), 7.06 (d, *J* = 8.4 Hz, 2H, *H*-2', *H*-6'), 6.21 (d, *J* = 2.6 Hz, 1H, *H*-13α), 5.54 (d, *J* = 2.6 Hz, 1H, *H*-13β), 4.78–4.80 (m, 1H, *H*-8), 3.16–3.18 (m, 1H, *H*-7), 2.42 (t, *J* = 7.6 Hz, 2H, *H*-3), 2.26–2.35 (m, 2H, overlapped, H_{eq}-6, H_{eq}-9), 1.93 (s, 3H, *H*-15), 1.56–1.69 (m, 2H, *H*-2), 1.11 (s, 3H, *H*-14), 0.92–1.03 (m, 2H, overlapped, H_{eq}-6, H_{eq}-9), 0.48–0.52 (m, 1H, *H*-1), 0.39–0.43 (m, 1H, *H*-5); ^{13}C NMR (125 MHz, $CDCl_3$) *δ*: 170.7 (*C*-12), 150.7 (*C*-4), 150.5 (*C*-4'), 139.6 (*C*-1'), 139.1 (*C*-11), 126.1 (*C*-2', *C*-6'), 122.7 (*C*-13), 111.6 (*C*-3', *C*-5'), 75.8 (*C*-8), 38.9 (*C*-3), 37.6 (*C*-7), 37.3 (*C*-9), 34.3 (*C*-1), 30.7 (*C*-6), 26.0 (*C*-2), 23.0 (*C*-5), 18.4 (*C*-14), 17.2 (*C*-10), 15.0 (*C*-15); H*R*-MS (ESI): *m*/*z* calcd for $C_{21}H_{26}N_3O_4$($[M + H]^+$), 384.1918; found, 384.1917.

24. ***8e****: (4aS,5S,5aR)-5a-methyl-3-methylene-5-(3-(2-(2-nitrophenyl)hydrazono)butyl)octahydro-2H-cyclopropa[f]benzofuran-2-one*

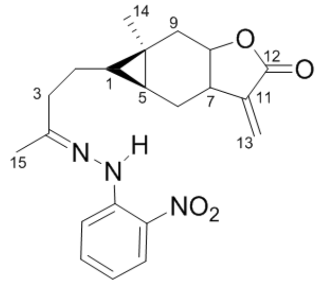

Red solid, mp: 89–90 ℃ ; ^{1}H NMR (500 MHz, $CDCl_3$) δ: 10.67 (s, 1H, NH), 8.15 (d, *J* = 8.7 Hz, 1H, *H*-3'), 7. 51–7.75 (m, 2H, *H*-4', *H*-5'), 7.49 (t, *J* = 7.8, 7.8 Hz, 1H, *H*-4'), 6.77 (d, *J* = 8.0 Hz, 1H, *H*-6'), 6.22 (d, *J* = 2.6 Hz, 1H, *H*-13α), 5.52 (d, *J* = 2.6 Hz, 1H, *H*-13β), 4.76–4.79 (m, 1H, *H*-8), 3.15–3.17 (m, 1H, *H*-7), 2.48 (t, *J* = 7.7, 7.7 Hz, 2H, *H*-3), 2.28–2.38 (m, 2H, overlapped, H_{eq}-6, H_{eq}-9), 2.01 (s, 3H, *H*-15), 1.52–1.78 (m, 2H, *H*-2), 1.12 (s, 3H, *H*-14), 0.92–1.01 (m, 2H, overlapped, H_{eq}-6, H_{eq}-9), 0.50–0.55 (m, 1H, *H*-1), 0.37–0.42 (m, 1H, *H*-5); ^{13}C NMR (125 MHz, $CDCl_3$) *δ*: 170.5 (*C*-12), 152.8 (*C*-4), 142.6 (*C*-2'), 139.1 (*C*-11), 136.2 (*C*-1'), 130.7 (*C*-5'), 125.9 (*C*-3'), 122.5 (*C*-13), 117.5 (*C*-4'), 115.8 (*C*-6'), 75.6 (*C*-8), 39.0 (*C*-3), 37.7 (*C*-7), 37.4 (*C*-9), 34.6 (*C*-1), 30.8 (*C*-6), 26.1 (*C*-2), 23.0 (*C*-5), 18.4 (*C*-14), 17.2 (*C*-10), 15.8 (*C*-15); H*R*-MS (ESI): *m*/*z* calcd for $C_{21}H_{26}N_3O_4$($[M + H]^+$), 384.1918; found, 384.1917.

*25. **8f**: (4aS,5S,5aR)-5a-methyl-3-methylene-5-(3-(2-(4-(trifluoromethyl)phenyl)hydrazono)butyl) octahydro-2H-cyclopropa[f]benzofuran-2-one*

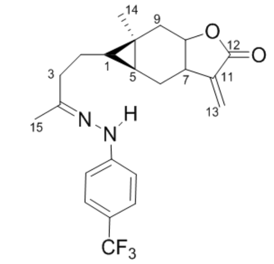

Brown solid, mp: 67–68 ℃ ; ^{1}H NMR (500 MHz, $CDCl_3$) δ: 7.42 (d, *J* = 8.3 Hz, 2H, *H*-2', *H*-6'), 7.09 (d, *J* = 8.3 Hz, 2H, *H*-3', *H*-5'), 6.18 (d, *J* = 2.6 Hz, 1H, *H*-13α), 5.49 (d, *J* = 2.6 Hz, 1H, *H*-13β), 4.74–4.76 (m, 1H, *H*-8), 3.11–3.13 (m, 1H, *H*-7), 2.39 (t, *J* = 7.6 Hz, 2H, *H*-3), 2.23–2.31 (m, 2H, overlapped, H_{eq}-6, H_{eq}-9), 1.86 (s, 3H, *H*-15), 1.51–1.67 (m, 2H, *H*-2), 1.08 (s, 3H, *H*-14), 0.86–0.98 (m, 2H, overlapped, H_{eq}-6, H_{eq}-9), 0.45–0.50 (m, 1H, *H*-1), 0.34–0.39 (m, 1H, *H*-5); ^{13}C NMR (125 MHz, $CDCl_3$) *δ*: 170.7 (*C*-12), 153.0 (*C*-4), 148.6 (*C*-1'), 148.1 (*C*-7'), 139.2 (*C*-11), 126.4 (*C*-3', *C*-5'), 122.8 (*C*-4'), 122.5 (*C*-13), 112.2 (*C*-2', *C*-6'), 75.9 (*C*-8), 38.8 (*C*-3), 37.6 (*C*-7), 37.3 (*C*-9), 34.5 (*C*-1), 30.8 (*C*-6), 26.1 (*C*-2), 22.9 (*C*-5), 18.2 (*C*-14), 17.1 (*C*-10), 14.8 (*C*-15); H*R*-MS (ESI): *m*/*z* calcd for $C_{22}H_{26}F_3N_2O_2$([M + H]$^+$), 407.1941; found, 407.1932.

*26. **8g**: (4aS,5S,5aR)-5a-methyl-3-methylene-5-(3-(2-(2,3,5,6-tetrafluorophenyl)hydrazono)butyl) octahydro-2H-cyclopropa[f]benzofuran-2-one*

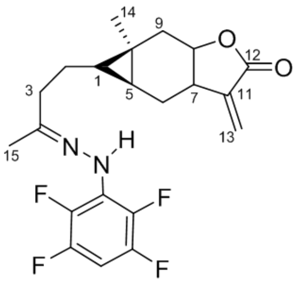

Brown ropy liquid; ^{1}H NMR (500 MHz, $CDCl_3$) δ: 6.78 (s, 1H, Ar-H), 6.20 (d, *J* = 2.5 Hz, 1H, *H*-13α), 5.55 (d, *J* = 2.5 Hz, 1H, *H*-13β), 4.76–4.81 (m, 1H, *H*-8), 3.16–3.20 (m, 1H, *H*-7), 2.46 (t, *J* = 7.6 Hz, 2H, *H*-3), 2.26–2.33 (m, 2H, overlapped, H_{eq}-6, H_{eq}-9), 1.94 (s, 3H, *H*-15), 1.49–1.69 (m, 2H, *H*-2), 1.10 (s, 3H, *H*-14), 0.88–1.00 (m, 2H, overlapped, H_{eq}-6, H_{eq}-9), 0.45–0.54 (m, 1H, *H*-1), 0.36–0.42 (m, 1H, *H*-5); ^{13}C NMR (125 MHz, $CDCl_3$) *δ*: 170.6 (*C*-12), 153.9 (*C*-4), 147.4 (*C*-3', *C*-5'), 145.4 (*C*-2', *C*-6'), 139.2 (*C*-11), 126.7 (*C*-1'), 122.3 (*C*-13), 96.3 (*C*-4'), 75.7 (*C*-8), 38.5 (*C*-3), 37.6 (*C*-7), 37.2 (*C*-9), 34.4 (*C*-1), 30.7 (*C*-6), 25.9 (*C*-2), 22.9 (*C*-5), 18.0 (*C*-14), 17.1 (*C*-10), 14.7 (*C*-15); H*R*-MS (ESI): *m*/*z* calcd for $C_{21}H_{23}F_4N_2O_2$([M + H]$^+$), 411.1690; found, 411.1689.

27. ***8h****: (4aS,5S,5aR)-5-(3-(2-(5,6-dimethylthieno[2,3-d]pyrimidin-4-yl)hydrazono)butyl)-5a-methyl-3-methyleneoctahydro-2H-cyclopropa[f]benzofuran-2-one*

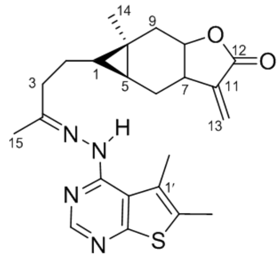

Light yellow solid, mp: 82–83 ℃ ; ^{1}H NMR (500 MHz, $CDCl_3$) δ: 8.42 (s, 1H, Ar-H), 6.21 (d, *J* = 2.5 Hz, 1H, *H*-13α), 5.53 (d, *J* = 2.5 Hz, 1H, *H*-13β), 4.76–4.81 (m, 1H, *H*-8), 3.12–3.18 (m, 1H, *H*-7), 2.51 (s, 3H, *H*-10’), 2.44 (t, *J* = 7.6 Hz, 2H, *H*-3), 2.40 (s, 3H, *H*-11’), 2.28–2.32 (m, 2H, overlapped, H_{eq}-6, H_{eq}-9), 2.07 (s, 3H, *H*-15), 1.52–1.72 (m, 2H, *H*-2), 1.09 (s, 3H, *H*-14), 0.93–1.03 (m, 2H, overlapped, H_{eq}-6, H_{eq}-9), 0.46–0.51 (m, 1H, *H*-1), 0.36–0.41 (m, 1H, *H*-5); ^{13}C NMR (125 MHz, $CDCl_3$) *δ*: 170.4 (*C*-12), 165.0 (*C*-1’), 157.4 (*C*-3’), 152.3 (*C*-4), 139.1 (*C*-11), 130.9 (*C*-8’), 130.8 (*C*-5’), 123.4 (*C*-7’), 122.5 (*C*-13), 116.8 (*C*-9’), 75.6 (*C*-8), 39.0 (*C*-3), 37.6 (*C*-7), 37.2 (*C*-9), 34.4 (*C*-1), 30.7 (*C*-6), 26.6 (*C*-2), 23.0 (*C*-5), 18.4 (*C*-14), 17.2 (*C*-10), 15.5 (*C*-15), 14.2 (*C*-11’), 13.2 (*C*-10’); H*R*-MS (ESI): *m*/*z* calcd for $C_{23}H_{29}N_4O_2S([M + H]^+)$, 425.2006; found, 425.2004.

28. ***8i****: 4-(2-(4-((4aS,5S,5aR)-5a-methyl-3-methylene-2-oxooctahydro-2H-cyclopropa[f]benzofuran-5-yl)butan-2-ylidene)hydrazinyl)benzoic acid*

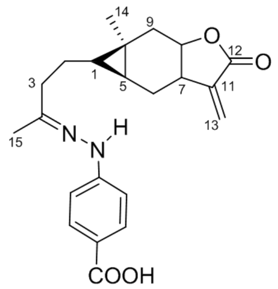

Brown solid, mp: 70–71 ℃ ; ^{1}H NMR (500 MHz, $(CD_3)_2CO$) δ: 10.86 (s, 1H, –COOH), 8.20 (d, *J* = 8.1 Hz, 2H, *H*-2’, *H*-6’), 7.85 (d, *J* = 8.1 Hz, 2H, *H*-3’, *H*-5’), 6.07 (d, *J* = 2.6 Hz, 1H, *H*-13α), 5.61 (d, *J* = 2.6 Hz, 1H, *H*-13β), 4.80–4.86 (m, 1H, *H*-8), 3.20–3.27 (m, 1H, *H*-7), 2.40 (t, *J* = 7.6 Hz, 2H, *H*-3), 2.24–2.33 (m, 2H, overlapped, H_{eq}-6, H_{eq}-9), 1.51–1.59 (m, 2H, *H*-2), 1.46 (s, 3H, *H*-15), 1.10 (s, 3H, *H*-14), 0.89–1.03 (m, 2H, overlapped, H_{eq}-6, H_{eq}-9), 0.52–0.58 (m, 1H, *H*-1), 0.40–0.45 (m, 1H, *H*-5); ^{13}C NMR (125 MHz, $(CD_3)_2CO$) *δ*: 170.7 (*C*-12), 150.7 (*C*-1’), 150.5 (*C*-4), 139.6 (*C*-4’), 139.1 (*C*-11), 126.1 (*C*-3’, *C*-5’), 122.7 (*C*-13), 111.6 (*C*-2’, *C*-6’), 75.8 (*C*-8), 38.9 (*C*-3), 37.6 (*C*-7), 37.3 (*C*-9), 34.3 (*C*-1), 30.7 (*C*-6), 26.0 (*C*-2), 23.0 (*C*-5), 18.4 (*C*-14), 17.2 (*C*-10), 15.0 (*C*-15); H*R*-MS (ESI): *m*/*z* calcd for $C_{22}H_{27}N_2O_4([M + H]^+)$, 383.1965; found, 383.1966.

© 2014 by the authors; licensee MDPI, Basel, Switzerland. This article is an open access article distributed under the terms and conditions of the Creative Commons Attribution license (http://creativecommons.org/licenses/by/3.0/).
